# Heterogeneous Nuclear Ribonucleoprotein F Mediates Insulin Inhibition of Bcl2-Modifying Factor Expression and Tubulopathy in Diabetic Kidney

**DOI:** 10.1038/s41598-019-43218-2

**Published:** 2019-04-30

**Authors:** Anindya Ghosh, Shuiling Zhao, Chao-Sheng Lo, Hasna Maachi, Isabelle Chenier, Muhammad Abdul Lateef, Shaaban Abdo, Janos G. Filep, Julie R. Ingelfinger, Shao-Ling Zhang, John S. D. Chan

**Affiliations:** 1 Département de medecine, Université de Montréal, Centre de recherche du Centre hospitalier de l’Université de Montréal (CRCHUM), 900 Saint Denis Street, Montréal, QC H2X 0A9 Canada; 2 Département de pathologie et biologie cellulaire, Université de Montréal, Centre de recherche, Hôpital Maisonneuve-Rosemont, 5415 boul. de l’Assomption, Montréal, QC H1T 2M4 Canada; 3 Harvard Medical School, Pediatric Nephrology Unit, Massachusetts General Hospital, 15 Parkman Street, WAC 709, Boston, MA 02114-3117 USA

**Keywords:** Transcriptional regulatory elements, Nephrons, Chronic kidney disease

## Abstract

We investigated the molecular mechanism(s) by which insulin prevents Bcl2-modifying factor (Bmf)-induced renal proximal tubular cell (RPTC) apoptosis and loss in diabetic mice. Transgenic mice (Tg) mice specifically overexpressing human BMF in RPTCs and non-Tg littermates were studied at 10 to 20 weeks of age. Non-diabetic littermates, diabetic Akita mice +/− insulin implant, Akita Tg mice specifically overexpressing heterogeneous nuclear ribonucleoprotein F (hnRNP F) in their RPTCs and immortalized rat renal proximal tubular cells (IRPTCs) were also studied. *BMF*-Tg mice exhibited higher systolic blood pressure, urinary albumin/creatinine ratio, RPTC apoptosis and urinary RPTCs than non-Tg mice. Insulin treatment in Akita mice and Akita mice overexpressing *hnRNP F* suppressed *Bmf* expression and RPTC apoptosis. In hyperinsulinemic-euglycemic wild type mice, renal *Bmf* expression was down-regulated with up-regulation of *hnRNP F*. *In vitro*, insulin inhibited high glucose-stimulation of *Bmf* expression, predominantly via p44/42 mitogen-activated protein kinase (MAPK) signaling. Transfection of *p44/42 MAPK* or *hnRNP F* small interfering RNA (siRNA) prevented insulin inhibition of *Bmf* expression. HnRNP F inhibited *Bmf* transcription via hnRNP F-responsive element in the *Bmf* promoter. Our results demonstrate that hnRNP F suppression of *Bmf* transcription is an important mechanism by which insulin protects RPTCs from apoptosis in diabetes.

## Introduction

Although glomerulopathy is a hallmark of early injury in diabetic kidney disease (DKD), tubulopathy including tubular atrophy and tubulointerstitial fibrosis is a major feature of later stages, closely associated with loss of renal function^1–4^. Therefore, tubulopathy is a better predictor of disease progression than glomerular damage^5–7^. Indeed, 71% of glomeruli from proteinuric diabetic patients were found to be attached to atrophic tubules at the glomerulotubular junction, including 8–17% atubular glomeruli (glomeruli without tubular attachment)^8,9^. The mechanisms underlying tubular atrophy are incompletely understood. One mechanism is apoptosis, which has been implicated in the subsequent loss of various renal cells, including renal proximal tubular cells (RPTCs)^10–15^. Thus, tubular apoptosis may precede tubular atrophy.

Hyperglycemia, hyperlipidemia, oxidative stress and dysfunction of the intrarenal renin-angiotensin system (RAS) have all been implicated in the progression of DKD. We documented that reactive oxygen species (ROS) mediate high glucose (HG) stimulation of angiotensinogen (Agt, the sole precursor of all angiotensins) gene expression in RPTCs *in vitro*^16,17^. Hyperglycemia and Agt act in concert to induce hypertension and RPTC apoptosis in type 1 diabetic (T1D) *Agt*-Tg mice^18,19^. Conversely, catalase (CAT) overexpression attenuates RPTC apoptosis in T1D *CAT*-Tg mice^20^ and T2D db/db *CAT*-Tg mice^21^, supporting the view that hyperglycemia via enhanced ROS generation plays a central role in RPTC apoptosis in diabetes.

We used microarray analysis to identify the downstream target genes of ROS, and noted elevated expression of Bcl2 (B-cell lymphoma 2)-modifying factor (Bmf) gene in the RPTCs of db/db mice; the elevated expression was, however, normalized in db/db *CAT*-Tg mice^22^. *In vitro*, Bmf overexpression enhanced and knockdown with small interference RNA (siRNA) decreased RPTC apoptosis in HG milieu. Furthermore, Bmf expression is markedly enhanced and localized to apoptotic RPTCs in human diabetic kidneys^22^. However, whether overexpression of Bmf would induce RPTC apoptosis and kidney injury *in vivo* has not been investigated.

Intensive insulin therapy has proven to be the most effective treatment for preventing nephropathy progression in T1D; however, the underlying mechanisms remain incompletely understood^23,24^. We previously reported that insulin inhibits high-glucose stimulation of renal rat *Agt* gene expression and RPTC hypertrophy through a putative insulin-responsive element (IRE) in the rat *Agt* gene promoter that interacts with 2 nuclear proteins, heterogeneous nuclear ribonucleoprotein F and K (hnRNP F/K) *in vitro*^25–27^. Overexpression of hnRNP F inhibited renal *Agt* gene expression and attenuated hypertension, kidney hypertrophy and RPTC apoptosis in Akita (T1D) and db/db Tg mice^28,29^. We further reported that hnRNP F/K mediate insulin inhibition of renal *Agt* gene expression and that insulin stimulates hnRNP F/K expression through p44/42 MAPK signaling pathway but not through the phosphatidylinositol 3-kinase (PI-3K) pathway in diabetic mice^30,31^. These findings suggest that insulin inhibition of renal *Agt* gene transcription and RPTC apoptosis occurs via hnRNP F/K in diabetes.

In the present study, we investigated the impact of Bmf overexpression on RPTC apoptosis in *BMF*-Tg mice and examined whether hnRNP F mediates, at least in part, insulin regulation of Bmf actions in Akita mice and in rat immortalized RPTCs (IRPTCs) cultured in HG milieu.

## Results

### RPTC-Specific Expression of Human Bcl2 Modifying Factor (*hBMF*) Transgene in Transgenic Mice

**Figure 1:**
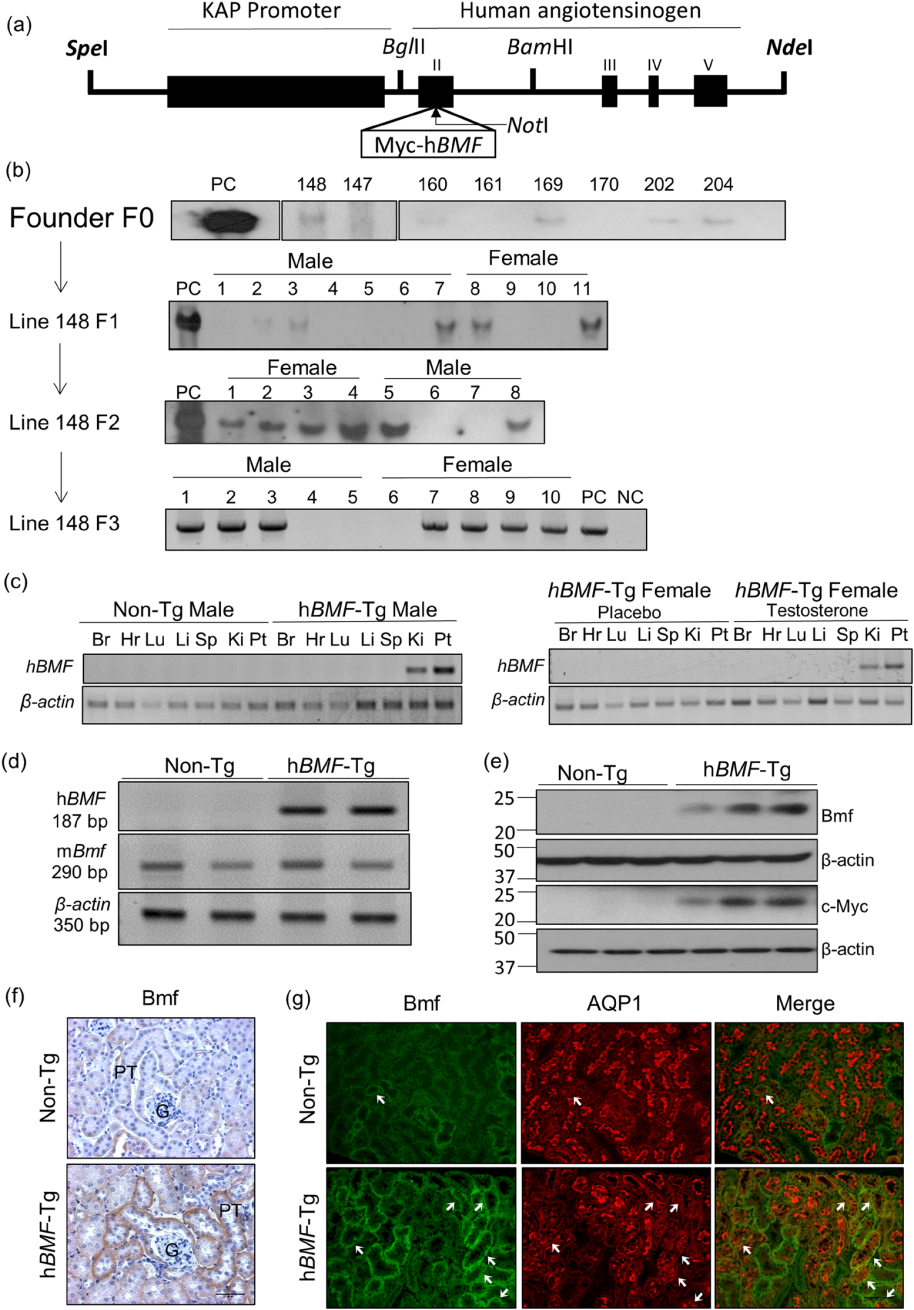
Generation of *hBMF*-Tg mice. (**a**) Schematic map of the kidney androgen-regulated promoter (KAP2)-Myc-*hBMF* construct. The isolated 17-kb KAP2-myc-*hBMF* transgene (digested with *Spe*I and *Nde*I) was microinjected into 1-cell fertilized mouse embryos obtained from super ovulated C57Bl6 C3H mice. (**b**) Southern blotting of genomic DNA for founders with biotin-labeled *BMF* probe. Heterozygous and homozygous F_1_, F_2_ and F_3_ were screened by PCR with specific primers (Table 1). Pc, plasmid positive control. NC, negative control. (**c**) RT-PCR product showing tissue expression of *hBMF* mRNA in male and in female Tg mice un-induced or induced with testosterone. *hBMF* and β-*actin* fragments are indicated. Female transgenic mice (line #148) mice were induced with placebo pellets or pellets containing 5 mg testosterone with a 21-day release schedule (Cat. #A-121, Innovative Research of America, Sarasota, FL) for 2 weeks prior to RNA isolation. Br, brain; Hr, heart; Lu, lung; Li, liver; Sp, spleen; Ki, kidney; PT, isolated proximal tubule. (**d**) Specific PCR analysis of *hBMF* transgene and mouse Bmf in offspring of non-Tg and *hBMF-*Tg line 148. (**e**) WB of Bmf and c-Myc protein expression in non-Tg and *hBMF-*Tg mice. (**f**) Representative immunostaining for Bmf expression in non-Tg and *hBMF-*Tg mice. (**g**) Representative colocalization of immunostaining for Bmf and aquaporin 1 (AQP1) in male non-Tg and *hBMF*-Tg mouse kidneys (x200). P: proximal tubule, G: glomerulus. Scale bar = 50 µm.

*hBMF-*Tg mice were generated by inserting *myc-hBMF* cDNA with the stop codon into a pKAP2 plasmid containing the kidney-specific androgen-regulated protein (KAP) promoter (Fig. 1a). Southern blot analysis revealed the presence of the transgene in heterozygote and homozygote animals from line 148 (Fig. 1b). Tissue-specific analysis by RT-PCR confirmed *hBMF* mRNA expression in the kidney cortex and RPTs isolated from male *hBMF-*Tg mice, as well as in the kidney cortex of female *hBMF-*Tg mice implanted with testosterone pellet but not in other tissues from both male and female mice (Fig. 1c). *hBMF* transgene was detected in RPTs of male *hBMF-*Tg mice but not in non-Tg mice and can be differentiated from endogenous mouse *Bmf* using primers specific for *hBMF* and mouse *Bmf* in RT-PCR, respectively (Fig. 1d). WB of isolated RPTs with anti-Bmf or anti-cMyc antibody (Fig. 1e) and immunostaining of kidney sections with an anti-Bmf antibody (that recognizes both human and mouse Bmf) (Fig. 1f) confirmed significantly higher BMF expression in RPTCs from male *hBMF-*Tg mice than in non-Tg mice. Furthermore, immunofluorescence staining with anti-Bmf and anti-aquaporin-1 (APQ1, a proximal tubule marker) revealed RPTC-specific BMF expression and its co-localization with AQP1-positive RPTCs of male *hBMF-*Tg mice (Fig. 1g and Supplementary Fig. 1c). These data demonstrate that hBMF expression is RPTC-specific in *hBMF-*Tg mice.

### Overexpression of hBMF Increases Systolic Blood Pressure and Kidney Injury in Tg Mice

**Figure 2:**
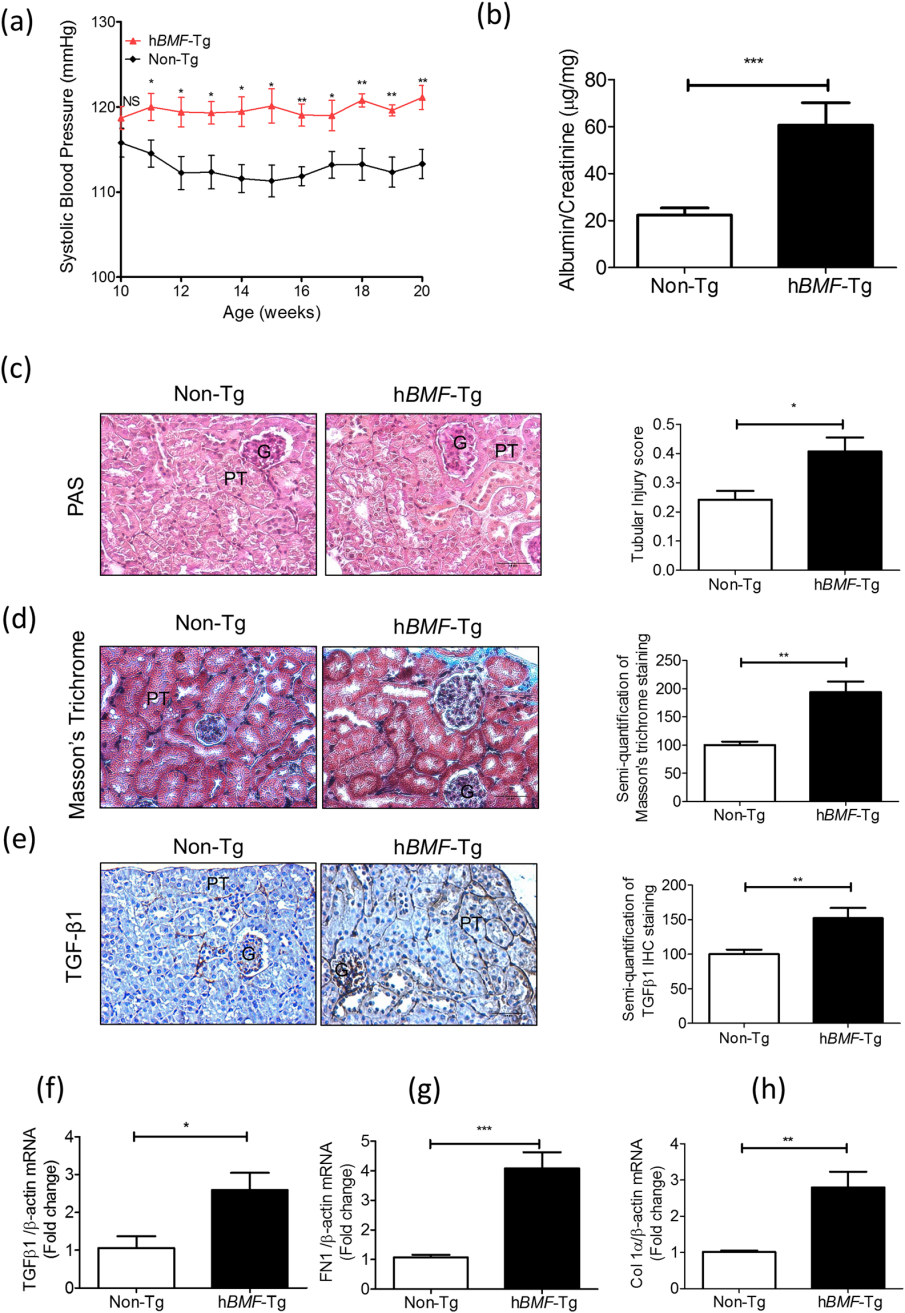
BMF overexpression induces systemic hypertension, glomerulo-tubular fibrosis and pro-fibrotic gene expression in Tg mouse kidneys at week 20. (**a**) Longitudinal changes in SBP (measured 2 to 3 times per mouse per week in the morning without fasting). Baseline SBP was recorded daily over 5 days before initiation of measurements. (**b**) Urinary albumin/creatinine ratio (ACR) in non-Tg and *hBMF-*Tg mice. (**c**) PAS staining and tubular injury score. (**d**) Masson’s trichrome staining and semi-quantification of staining. (**e**) TGF-β1 immunostaining and semi-quantification in non-Tg and *hBMF-*Tg mice. Scale bar = 50 µm. RT-qPCR of *TGF-β1* (**f**), *FN1* (**g**) and *Col Iα* (h) mRNA in freshly-isolated RPTs from non-Tg and *hBMF*-Tg mice. Values are means ± SEM, n = 6. *p < 0.05; **p < 0.01; ***p < 0.005; NS, Not significant.

Longitudinal systolic blood pressure (SBP) measurements revealed consistently higher SBP in *hBMF-*Tg mice than in non-Tg mice from week 11 to 20 (Fig. 2a). Cross-sectional SBP measurements at week 20 showed significantly elevated SBP (on average by ~8 mm Hg) in *hBMF-*Tg mice as compared with non-Tg mice (Table 1).

**Table 1:** Physiological Measurements.

	Non-Tg	h*BMF*-Tg
Blood glucose (BG, mM) (n = 10)	9.8 ± 0.8	10.16 ± 1.12
Systolic blood pressure (SBP, mmHg) (n = 10)	113.57 ± 1.3	121.12 ± 1.4**
Body weight (BW, g) (n = 10)	34.76 ± 1.47	33,77 ± 1.08
Kidney weight (KW, mg) (n = 10)	363.0 ± 23.3	340.8 ± 16.07
KW/BW (mg/g) (n = 10)	10.38 ± 0.41	10.15 ± 0.47
Tibia length (TL, mm) (n = 10)	21.75 ± 0.33	20.80 ± 0.3
KW/TL (mg/mm) (n = 10)	16.65 ± 0.97	16.50 ± 0.61
GFR/BW(ml/min/g) (n = 10)	7.8 ± 0.37	9.8 ± 0.32*
Glomerular tuft volume (*10^3^ µm^3^) (n = 6)	139.6 ± 5.6	166.7 ± 7.24**
Tubular luminal area (µm^2^) (n = 6)	47.46 ± 2.39	48.19 ± 3.1
RPTC volume (*10^3^ µm^3^) (n = 6)	7.74 ± 0.36	9.63 ± 0.4**
ACR (µg/mg) (n = 13)	22.37 ± 2.95	60.62 ± 9.53***

*p < 0.05, **p < 0.01, ***p < 0.001 vs Non-Tg.

Marked increases in urinary albumin/creatinine ratio (ACR) were observed in *hBMF*-Tg mice at 20 weeks of age (Fig. 2b and Table 1). Blood glucose, body weight (BW), kidney weight (KW), tibial length (TL), BW/KW, or BW/TL did not differ significantly between *hBMF-*Tg mice and non-Tg mice (Table 1). Periodic acid Schiff (PAS) staining of kidney sections revealed mild structural changes in *hBMF-*Tg compared to non-Tg mice and yielded higher statistically significant tubular injury scores (Fig. 2c). Moreover, more pronounced Masson’s Trichrome staining and TGFβ1 immunostaining were detected in glomerulo-tubular areas of *hBMF*-Tg mice as compared to non-Tg controls and confirmed by semi-quantification (Fig. 2d,e, respectively). RT-qPCR showed significantly increased mRNA levels of TGFβ1 (Fig. 2f), fibronectin (Fn1) **(**Fig. 2g**)** and collagen 1α (Col 1α) **(**Fig. 2h**)** in RPTs of *hBMF-*Tg mice compared to non-Tg mice, indicating that BMF overexpression induces tubulointerstitial fibrosis in kidneys of *hBMF-*Tg mice. Moreover, morphological measurements revealed significant increases in glomerular tuft volume and RPTC volume in *hBMF-*Tg mice as compared to non-Tg mice (Table 1).

### Overexpression of BMF Stimulates RPTC Apoptosis and Increases Urinary RPTCs

**Figure 3:**
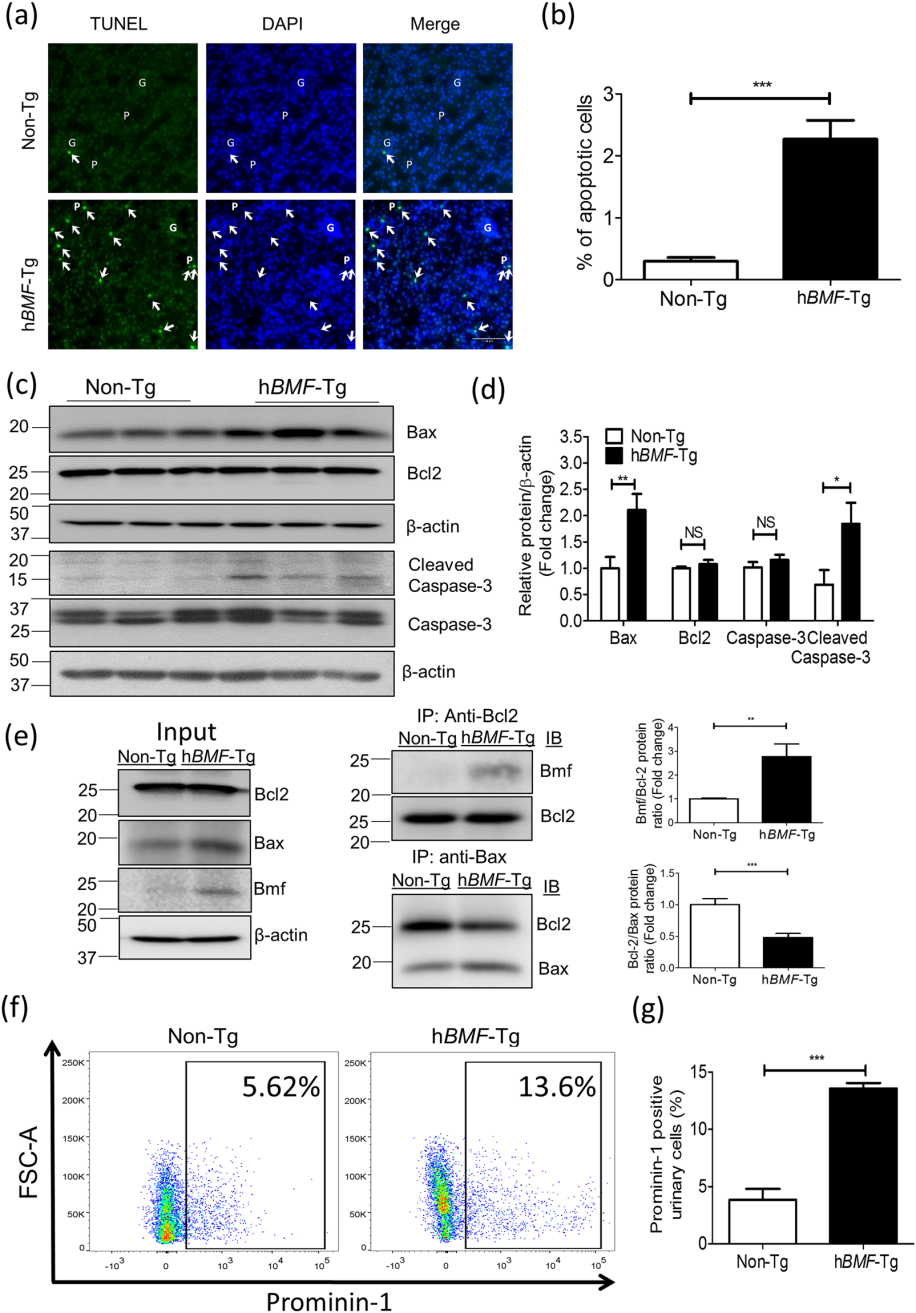
Overexpression of BMF induces RPTC apoptosis in mouse kidneys at age 20 weeks. (**a**) TUNEL (green fluorescence) Assay. Magnification x200. Arrowheads indicate apoptotic cells. P: proximal tubule, G: glomerulus. Scale bar = 50 µm. (**b**) Semi-quantitation of apoptotic cells in mouse kidneys. (**c**) WB of Bax, Bcl-2, caspase-3 and cleaved (c)-caspase-3, in freshly-isolated RPTs from non-Tg and *hBMF-*Tg mice and (**d**) densitometry analysis. Values are means + SEM, n = 6. **p < 0.01; ***p < 0.005; NS, Not significant. (**e**) Co-immunoprecipitation of Bcl-2 with Bmf and Bax with Bcl2 in RPT extracts from non-Tg and h*BMF-*Tg mice. The relative densities of bands of co-IP of Bmf/Bcl2 and Bcl2/Bax were quantified by NIH ImageJ software (http://rsb.info.nih.gov/ij/). (**f**) Analysis of urinary prominin-1 positive RPTCs in non-Tg and *hBMF-*Tg mice by flow cytometry. (**g**) Quantitation of urinary RPTCs/total urinary cells in non-Tg and *hBMF-*Tg mice. Values are means ± SEM, n = 3. ***p < 0.005; NS, Not significant.

The percentage of TUNEL-positive cells was significantly higher in *hBMF-*Tg mice as compared to non-Tg mice (Fig. 3a,b). WB analysis showed increases in Bax and cleaved caspase-3 expression (Fig. 3c,d) without detectable changes in Bcl2 and caspase-3 expression in RPT extracts of *hBMF-*Tg mice as compared to non-Tg mice. Furthermore, co-immunoprecipitation (co-IP) experiments revealed more BMF co-IP with Bcl2 whereas less Bcl2 co-IP with Bax in RPTs of *hBMF*-Tg mice than non-Tg mice (Fig. 3e). These data would indicate that in *hBMF-*Tg mice, BMF predominantly binds Bcl2 and dissociates it from Bax, thereby tipping the balance of the Bax/Bcl2 ratio towards caspase-3 activation and subsequently RPTC apoptosis.

As Bmf is known to enhance anoikis^32,33^, we investigated whether overexpression of BMF would increase the number of RPTCs in the urine as an indirect indicator of loss of RPTCs. Using flow cytometry, we detected a greater percentage of cells in the urine that stained positive for prominin-1, a proximal tubule marker^34^ in *hBMF-*Tg than in non-Tg mice (Fig. 3f,g). These data indicate that overexpression of BMF induces RPTC loss, followed by shedding into the urine in *hBMF-*Tg mice.

### Insulin Inhibits Bmf Expression and RPTC Apoptosis in Akita Mice

**Figure 4:**
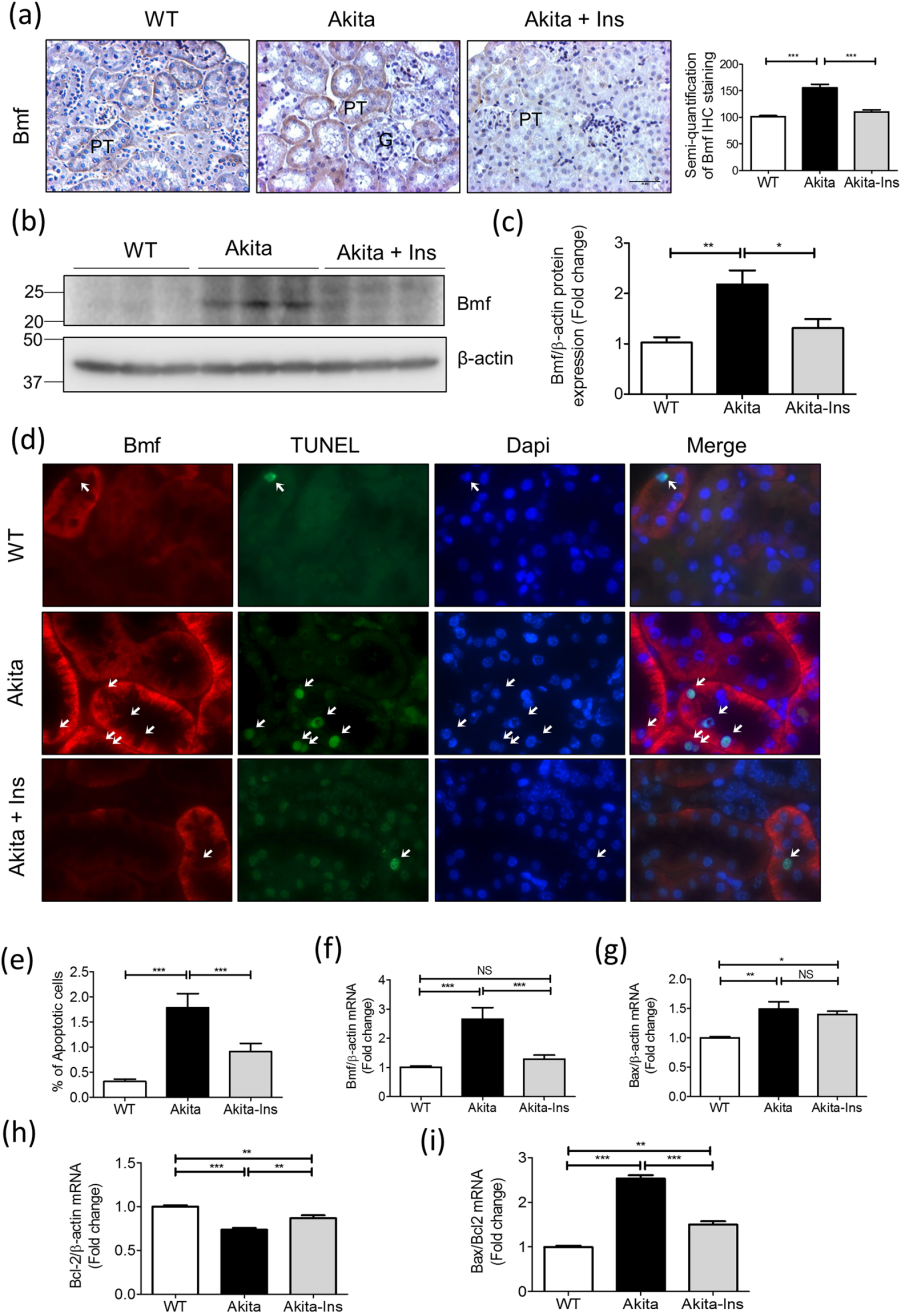
Insulin effect on Bmf expression and RPTC apoptosis in Akita mice at week 16. (**a**) Bmf immunostaining (magnification 200x) and semi-quantification of relative Bmf immunostaining by NIH Image J software (http://rsb.info.nih.gov/ij/). (**b**) Representative WB of Bmf and (**c**) densitometry analysis in freshly-isolated RPTs from WT, Akita and insulin-treated Akita mice. (**d**) Co-localization of Bmf expression and TUNEL-positive cells in Akita mouse kidneys. Kidney sections were subjected to TUNEL assay to visualize apoptotic cells (green) and then incubated with anti-Bmf antibody followed by anti-goat AlexaFluor 594 to demonstrate Bmf expression (red). Magnification 600X. Arrows indicate cells that stained positively for TUNEL and Bmf. G, glomerulus; and PT, proximal tubule. (**e**) Semi-quantitation of apoptotic cells in WT, Akita and insulin-treated Akita mice. RT-qPCR of Bmf (**f**), Bax (**g**), Bcl-2 (**h**) and ratio of Bax/Bcl-2 (**i**) mRNA in freshly isolated RPTs from WT, Akita and insulin-treated Akita mice. Values are means ± SEM, n = 6. *p < 0.05; **p < 0.01; ***p < 0.005; NS, Not significant.

We next tested whether insulin reduction of RPTC apoptosis in Akita mice is mediated, at least in part, via suppression of Bmf expression. As anticipated, Bmf expression was higher in RPTs of Akita mice than in WT mice and was inhibited by insulin (Fig. 4a). WB for Bmf protein expression in isolated RPTs confirmed these findings (Fig. 4b,c). Co-immunostaining studies showed Bmf localization to TUNEL-positive RPTCs of Akita mice, which was normalized by insulin treatment (Fig. 4d). The percentage of TUNEL-positive RPTCs was significantly higher in Akita than in WT mice and was normalized by insulin treatment (Fig. 4e). RT-qPCR analysis revealed increases in Bmf (Fig. 4f) and Bax mRNA expression (Fig. 4g), with decreases in Bcl-2 mRNA (Fig. 4h) in Akita mice. These changes in the Akita mice were normalized by insulin treatment with the exception of Bax mRNA. Consistently, the Bax/Bcl-2 mRNA ratio was significantly increased in Akita mice and was reversed by insulin treatment (Fig. 4i).

### Insulin Inhibits Bmf Expression Independent of its Glucose Lowering Effect

**Figure 5:**
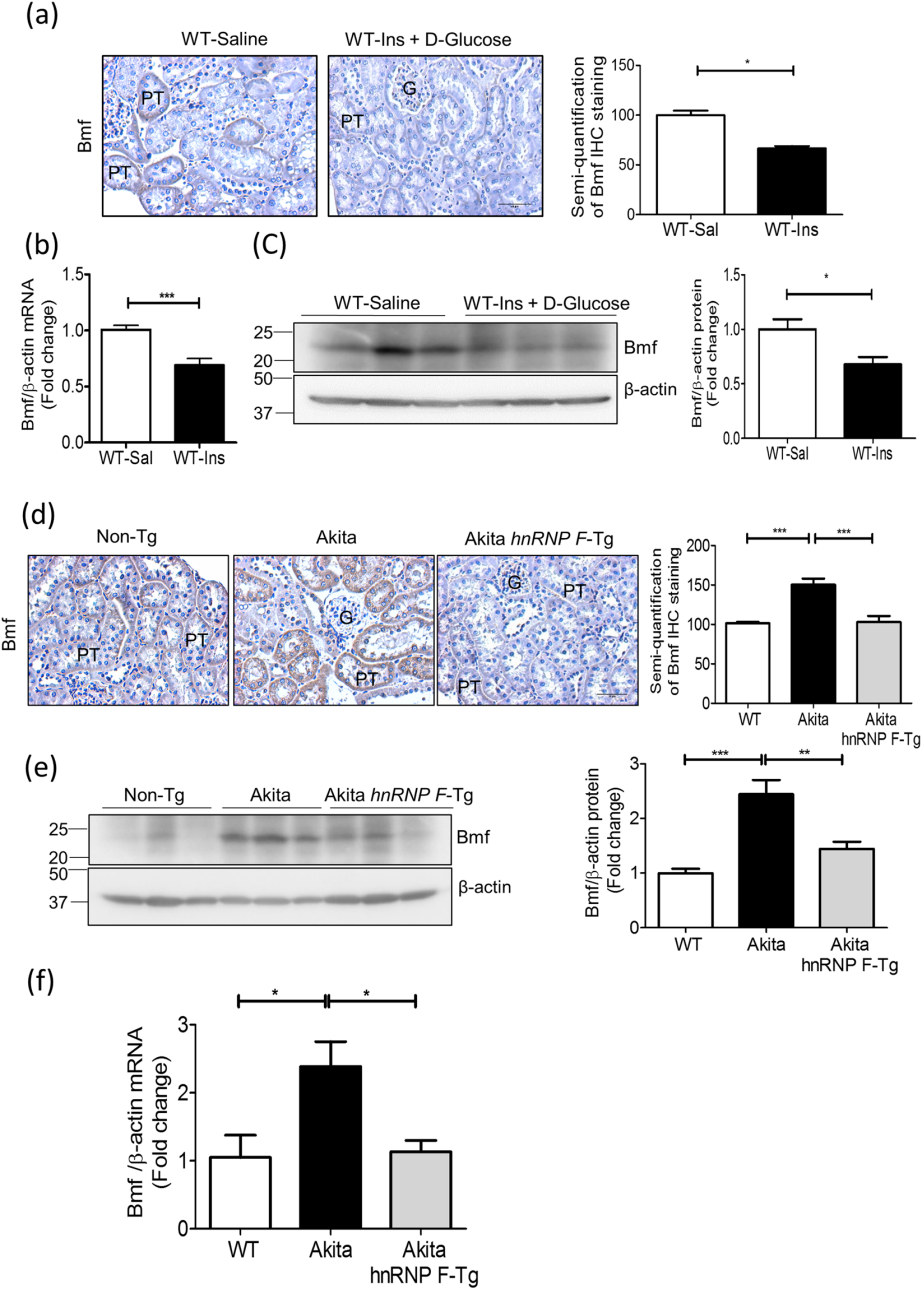
Renal Bmf expression in hyperinsulinemic-euglycemic mice and in Akita *hnRNP* F-Tg mice. (**a**) Representative immunostaining of Bmf (magnification X 200), (**b**) RT-qPCR of *Bmf* mRNA expression and (**c**) representative WB and densitometry of Bmf expression in isolated RPTs from WT mice after 3-h infusion with saline (open bars) or insulin (Ins) + D-glucose (solid black bars). Values are mean ± SEM, n = 8 per group. *p < 0.05; ***p < 0.005; NS, not significant. (**d**) Representative immunostaining for Bmf (magnification X 200). Semi-quantification of relative Bmf immunostaining in (**a,d**) was assessed by NIH Image J software (http://rsb.info.nih.gov/ij/), (**e**) Representative Bmf WB and densitometry analysis, and (**f**) RT-qPCR of *Bmf* mRNA expression in isolated RPTs from WT, Akita and Akita *hnRNP* F-Tg mice at 20 weeks of age. Values are mean ± SEM, n = 8 per group. *p < 0.05; **p < 0.01; NS, not significant.

We previously reported that hyperinsulinemia up-regulated hnRNP F expression in RPTCs independent of its glucose lowering effect^31^. To investigate whether insulin could also inhibit renal Bmf expression independent of its effect on lowering systemic blood glucose *in vivo*, hyperinsulinemic-euglycemic clamp experiments were performed on non-diabetic WT mice. Consistent with the previous report^31^, hyperinsulinemia resulted in increases in hnRNP F immunostaining (Supplementary Fig. 1a) and decreases in Bmf immunostaining (Fig. 5a) and Bmf mRNA (Fig. 5b) as compared with saline infusion. WB of Bmf expression (Fig. 5c) confirmed these observations, indicating that insulin suppression of renal *Bmf* expression occurs independently of its glucose lowering action.

### HnRNP F Overexpression Inhibits Bmf Expression in Akita *hnRNP F-*Tg Mice

To explore the functional relationship between hnRNP F and Bmf expression in diabetes, we compared Akita *hnRNP F-*Tg mice to non-Tg mice and Akita mice^28^. Significant decreases in hnRNP F immunostaining (Supplementary Fig. 1b) and increases in Bmf immunostaining were observed in kidneys of Akita mice as compared with non-Akita WT mice, whereas Bmf immunostaining was reduced in Akita *hnRNP F*-Tg mice (Fig. 5d). WB of Bmf protein expression (Fig. 5e) and RT-qPCR of *Bmf* mRNA expression (Fig. 5f) confirmed these observations. These data would imply a role for hnRNP F in mediating insulin suppression of *Bmf* expression.

### Insulin Inhibits *Bmf* Expression via HnRNP F in IRPTCs

**Figure 6:**
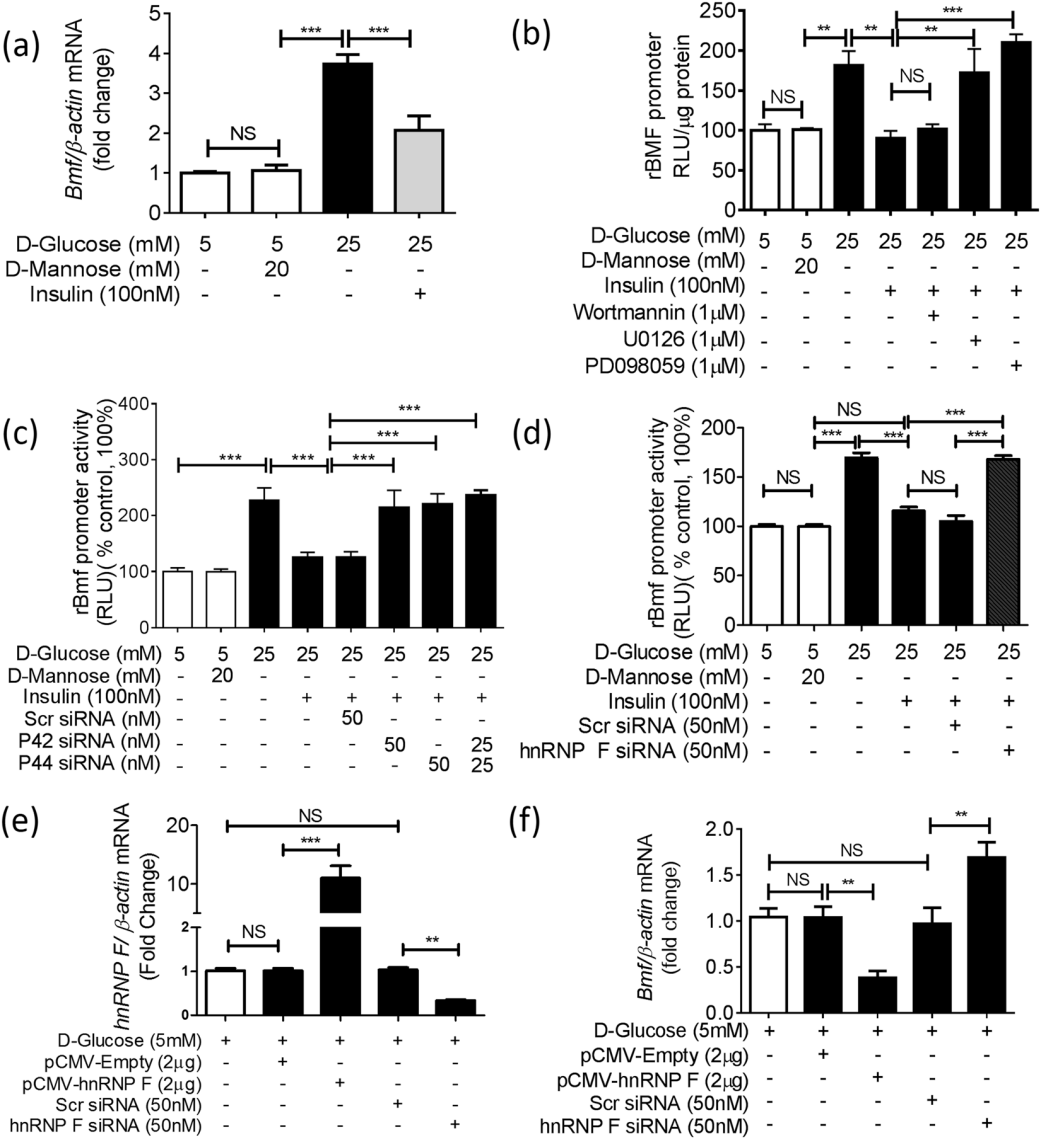
Effect of siRNA of p44/42 *MAPK* or *hnRNP F* and *hnRNP F* cDNA on *Bmf* gene expression in IRPTCs. (**a**) Bmf mRNA expression in IRPTCs cultured in NG or HG in the presence or absence of insulin. **(b)** IRPTCs stably transfected with pGL4.20-rat *Bmf* gene promoter were incubated in NG or HG DMEM ± insulin for 24 h with or without pharmacological inhibitors or transiently transfected with siRNA of p42 *MAPK* or p44 *MAPK* (**c**) or with siRNA of *hnRNP F* (**d**). IRPTCs transiently transfected with pCMV empty or pCMV-*hnRNP F* plasmid and scrambled (Scr) siRNA or siRNA of *hnRNP F* to determine *hnRNP F* mRNA (**e**) or *Bmf* mRNA (**f**) expression. Luciferase activity in cells cultured in NG medium was considered as 100%. The results are expressed as percentage of control (mean ± SEM, n = 3). Each value represents the mean ± SEM (n = 3) assayed in duplicate. **p < 0.01; ***p < 0.005; NS, not significant.

Confirming our previous findings^22^, we found that HG stimulated *Bmf* mRNA expression in cultured IRPTCs and that insulin reversed this finding (Fig. 6a). Insulin treatment also prevented the stimulatory effect of HG on rat *Bmf* promoter (N-1370/+102) activity (Fig. 6b). Pharmacological blockade of p44/42 MAPK (with PD98059 and U0126) effectively prevented insulin inhibition of *Bmf* promoter activity, whereas blockade of PI-3-Kinase with wortmannin was without effect (Fig. 6b). Moreover, transfection with siRNA of *p44 MAPK* or *p42MAPK* also abolished insulin inhibition of *Bmf* promoter activity in HG, whereas scrambled (Scr) siRNA had no effect (Fig. 6c).

Furthermore, siRNA of *hnRNP F*, but not Scr siRNA abolished insulin inhibition of *Bmf* promoter activity in HG (Fig. 6d). Consistently, we detected 10-fold increase and 70% decrease of hnRNP F mRNA expression when IRPTCs were transfected with pCMV-*hnRNP F-HA* plasmid and siRNA of *hnRNP F*, respectively (Fig. 6e). In contrast, Bmf mRNA expression decreased by 60% and increased 175% when IRPTCs were transfected with pCMV-*hnRNP F-HA* and *hnRNP F* siRNA as compared to controls, respectively (Fig. 6f).

### Identification of hnRNP F-*Response Element* (*RE*) in Rat *Bmf* Promoter

**Figure 7:**
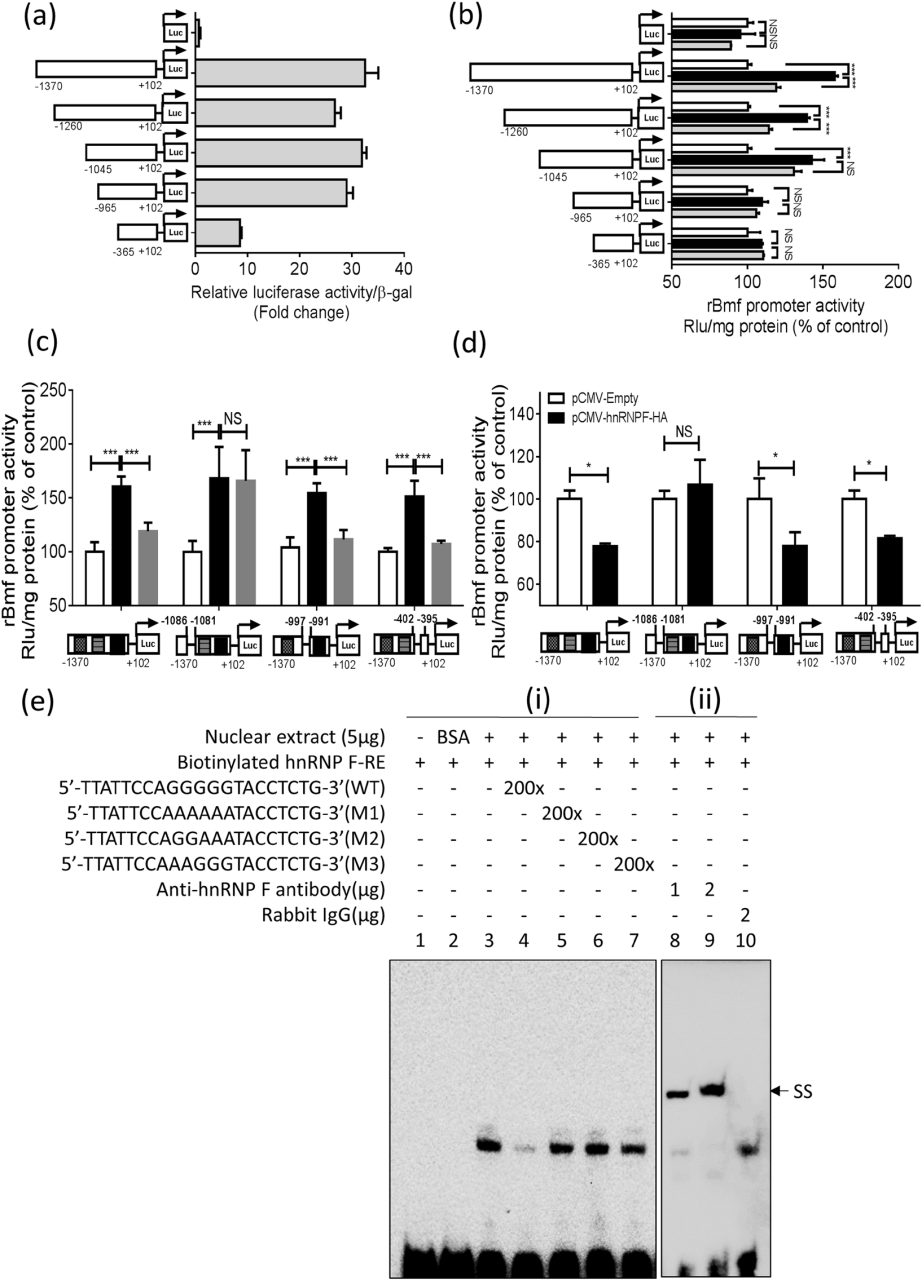
Identification of putative *IRE* or *hnRNP F-RE* in the *Bmf* gene promoter. (**a**) Luciferase activity of plasmids containing various lengths of *Bmf* gene promoter in NG medium or (**b)** HG medium ± insulin in IRPTCs. Luciferase activities were normalized by co-transfecting the vector pRC/RSV containing beta-galactosidase cDNA. Control IRPTCs in NG (open bars), IRPTCs in HG (solid black bars) and IRPTCs treated with insulin in HG (solid light grey). (**c**) The activity of 1 μg of the full-length *Bmf* gene promoter ± deletion of putative *IRE*(N-1086 to N-1081: 5′-AGGGGG-3′), (N-997 to N-991: 5′-GAGGGGC-3′) and (N-402to N-395: 5′-CCCCCGC-3′) in the *Bmf* gene promoter in IRPTCs in NG or HG medium ± insulin. (**d**) The activity of 1 μg of the full-length *Bmf* gene promoter with or without deletion of putative *IRE* transfected with *hnRNP F* cDNA in IRPTCs in NG medium. Values are mean ± SEM, n = 3. (*p < 0.05; ***p < 0.005 NS, not significant). (**e**) EMSA of putative biotinylated *IRE* (N-1093 to N-1072) with RPTC nuclear proteins with or without excess unlabeled WT *IRE* or mutated *IRE*. For supershift rabbit anti-hnRNP F or rabbit IgG was added to the reaction mixture and incubated for 30 minutes on ice before incubation with biotinylated probe. The results are representative of 3 independent experiments. SS, supershift band.

To identify putative hnRNP F-*RE* that mediate the inhibitory action of insulin on *Bmf* promoter activity, pGL4.20 plasmid containing different lengths of the rat *Bmf* promoter were transiently transfected into IRPTCs. pGL4.20-*Bmf* promoter (N-1370/N + 102), pGL4.20-*Bmf* promoter (N-1260/N + 102), pGL4.20-*Bmf* promoter (N-1045/N + 102), and pGL4.20-*Bmf* promoter (N-965/N + 102) displayed a 32-, 26-, 32- and 29-fold increase in activity compared with control pGL4.20 plasmid in IRPTCs in NG (Fig. 7a). Deletion of nucleotides N-1370 to N-365 significantly decreased pGL4.20-*Bmf* promoter (N-365/+102) activity to just 8-fold higher than plasmid pGL4.20 (Fig. 7a). Insulin prevented HG stimulation on pGL4.20-*Bmf* promoter (N-1370/N + 102) and pGL4.20-*Bmf* promoter (N-1260/N + 102) activity whereas insulin failed to affect pGL4.20-*Bmf* promoter (N-1045/N + 102), pGL4.20-*Bmf* promoter (N-965/N + 102) and pGL4.20-*Bmf* promoter (N-365/N + 102) activity (Fig. 7b).

Intriguingly, *Bmf* promoter with deletion of nucleotides N-1086 to N-1081, pGL4.20-*Bmf* promoter (N-1370/N + 102 ∆ N-1086/N-1081) completely abolished the response to insulin in HG as compared to pGL4.2-*Bmf* promoter (N-1370/N + 102) (Fig. 7c). In contrast, *Bmf* promoter with deletion of N-997 to N-991 and N-402 to N-395 did not alter the response to insulin (Fig. 7c). Furthermore, pGL4.20-*Bmf* promoter (N-1370/N + 102) activity was significantly suppressed when co-transfected with pCMV-*hnRNP F*-HA, whereas it had no detectable effect on pGL4.20-*Bmf* promoter (N-1370/N + 102 ∆ N-1086/N-1081) in NG (Fig. 7d). Moreover, *Bmf* promoter with deletion of nucleotides N-997 to N-991 and N-402 to N-395 did not affect the inhibitory effect of pCMV-*hnRNP F*-HA on *Bmf* promoter activity. These results point toward that nucleotides N-1086 to N-1081 is a putative core *RE* responding to insulin and hnRNP F.

Indeed, EMSA showed that the WT double-strand DNA fragment (N-1093 to N-1072) binds to IRPTC nuclear proteins, which could be displaced by the WT DNA fragment, but not by mutated DNA fragments (M1, M2 and M3) (Fig. 7e). Moreover, incubation with an anti-hnRNP F antibody, but not with rabbit IgG (control) induced a supershift (SS) of the *hnRNP F-RE* with nuclear proteins (Fig. 7e).

## Discussion

The present study demonstrates that overexpression of hBMF specifically in the RPTCs enhances RPTC apoptosis and loss into the urine in *hBMF-*Tg mice, indicating a critical role for Bmf in mediating tubular cell apoptosis and loss. Our findings also indicate that insulin treatment prevents RPTC apoptosis and inhibits renal *Bmf* transcription via a novel putative *insulin-response element* (*IRE*) in the *Bmf* gene promoter that interacts with hnRNP F. These findings identify a novel mechanism by which insulin may prevent nephropathy progression in diabetes.

Bmf is a member of the BH3-only protein family^32^. Bmf binds to cytoskeletal structures and is sequestered to myosin V motors through association with dynein light chain 2. Certain damage signals, such as the detachment of adherent cells (anoikis) from their substratum trigger the release of Bmf, which through binding to pro-survival Bcl-2 proteins induces dissociation of the Bcl-2/Bax dimer, thereby allowing the pro-apoptotic action of Bax on mitochondria^32,33,35,36^. Bmf is expressed in various organs, including the kidney^33^. Mice deficient in Bmf^−/−^, however, do not display any obvious phenotypic abnormalities^37,38^. Recent studies by Pfeiffer *et al*. demonstrated that Bmf not only plays a major role in progressive pancreatic β-cell death in HNF1*α*-MODY, but also contributes to the pancreatic beta-cell function to maintain glucose homeostasis, independent of cell death signaling^39^. However, little is known about the role of Bmf in the kidney or in the pathogenesis of diabetes. We previously reported the presence of increased Bmf expression in apoptotic RPTCs in db/db mice as well as in human diabetic kidneys^22^.

To provide direct evidence for Bmf-mediated RPTC apoptosis *in vivo*, we generated Tg mice specifically overexpressing hBMF in their RPTCs. Our data demonstrate that overexpression of hBMF in RPTCs indeed induces RPTC apoptosis and loss into the urine in *hBMF-*Tg mice. Furthermore, overexpression of hBMF increased expression of Bax and cleaved (active) caspase-3 and enhanced Bax binding to Bcl2, consistent with Bax-mediated activation of the intrinsic (mitochondrial) pathway of apoptosis.

The mechanisms underlying increased SBP and ACR in *hBMF-*Tg mice are incompletely understood. The possibility that up-regulation of *TGFβ1* gene expression in RPTCs and its downstream targets Fn1 and Col 1α, leading to higher tubulointerstitial fibrosis that facilitates the development of hypertension has received considerable attention. Indeed, a strong correlation was found between interstitial fibrosis and the development of SBP via a loss or rarefaction of capillaries around the tubules^40,41^. Our observations of increased glomerular tuft volume, RPTC volume and GFR in *hBMF-*Tg mice lend additional support to this notion. Furthermore, the observed increases in ACR can be explained, at least in part, by a combination of loss of RPTCs and increased GFR^42^.

The Akita mouse is an autosomal dominant spontaneous diabetic mouse model (mutation in *insulin2*) that exhibits many features resembling changes in T1D patients including hypoinsulinemia, hyperglycemia, hypertension and renal dysfunction^43,44^.

Insulin suppressed *Bmf* and stimulated *hnRNP F* expression in RPTCs of hyperinsulinemic-euglycemic mice after 3 h of hyperinsulinemia as compared to Akita mice after 4 weeks of insulin implantation. This rapid transcription is in agreement with previous reports that up-regulated and down-regulated genes in the skeletal muscle and liver were observed within 2 to 4 h under hyperinsulinemic-euglycemic condition^45,46^. These data demonstrate that insulin impacts RPT *Bmf* and *hnRNP F* expression, independent of its glucose-lowering effect.

Our data also revealed significantly higher expression of Bmf protein and mRNA in RPTs of 16 weeks old Akita than WT mice. Overexpression of hnRNP F significantly down-regulated *Bmf* expression in Akita *hnRNP F-*Tg mice, suggesting a role for hnRNP F in mediating insulin inhibition of *Bmf* expression in Akita mice. However, it is noteworthy that we could not exclude the off-target effects of random transgene insertion in mediating insulin suppression of Bmf expression in our hnRNP F-Tg mice. Addressing this issue would require mapping transgene insertion sites which is outside the scope of this present study. Generation of currently unavailable RPTC-specific hnRNP F knockout mice would provide more direct evidence of the role of hnRNP F on Bmf expression. We are now working on developing such a model for future work.

Combining studies with pharmacological inhibitors and siRNAs, we identified roles for the p44/42 MAPK signaling pathway and hnRNP F in mediating insulin suppression of renal *Bmf* gene transcription. These findings clearly link hnRNP F to mediating insulin inhibition of *Bmf* gene expression in the diabetic mouse kidney. Nevertheless, additional studies employing RPTC-specific *hnRNP F* knockout mice are needed to firmly establish this pathway.

The mechanism by which hnRNP F down-regulates renal *Bmf* gene expression remains to be investigated. A likely mechanism is that hnRNP F suppresses *Bmf* transcription via binding to a putative *IRE* in the *Bmf* promoter. This is supported by the findings that transfection of *hnRNP F* cDNA considerably decreases *Bmf* promoter activity, whereas transfection of *hnRNP F* siRNA reverses insulin effect. DNA sequence analysis revealed 3 GC-rich regions in the *Bmf* promoter including nucleotides N-1086 to N-1081 (5′-AGGGGG-3′), N-997 to N-991 (5′-GAGGGGC-3′) and N-402 to N-395 (5′-CCCCCGC-3′). Nucleotides N-1086 to N-1081 contain the sequence 5′-AGGGGG-3′ which is homologous to the *IRE* sequence of N-402 to N-398 (5′-AGGGGG-3′) and N-974 to N-969 (5′-AGGGGG-3′) in the rat *Sirtuin-1* and *Ace2* promoter, respectively^29,47^. Deletion of N-1086 to N-1081 in the *Bmf* promoter markedly reduced insulin- and hnRNP F-down-regulation of *Bmf* promoter activity in IRPTCs whereas deletion of N-997 to N-991 and N-402 to N-395 had no effect, indicating that the 5′- and 3′-flanking sequences of the core sequence might be important for hnRNP F binding. Moreover, biotinylated *IRE* (N-1093 to N-1072) binds to nuclear proteins of RPTCs. Addition of anti-hnRNP F antibody yielded a supershift of biotinylated *IRE* complex with nuclear proteins on EMSA. These findings identify N-1093 to N-1072 as a putative *IRE* that interacts with hnRNP F and inhibits *Bmf* transcription.

It is noteworthy that hnRNP F not only regulates *Bmf* transcription as it also modulates the transcription of *Agt*^28^, Sirtuin-1^29^, *Nrf2*^31^, *Ace2*^47^ and possibly other genes^48^. Thus, the hnRNP F effect on Bmf expression might also be mediated by other (indirect) mechanisms. Furthermore, the role of hnRNP F in human pathophysiology remains to be explored. More studies are definitely needed along these lines.

**Figure 8:**
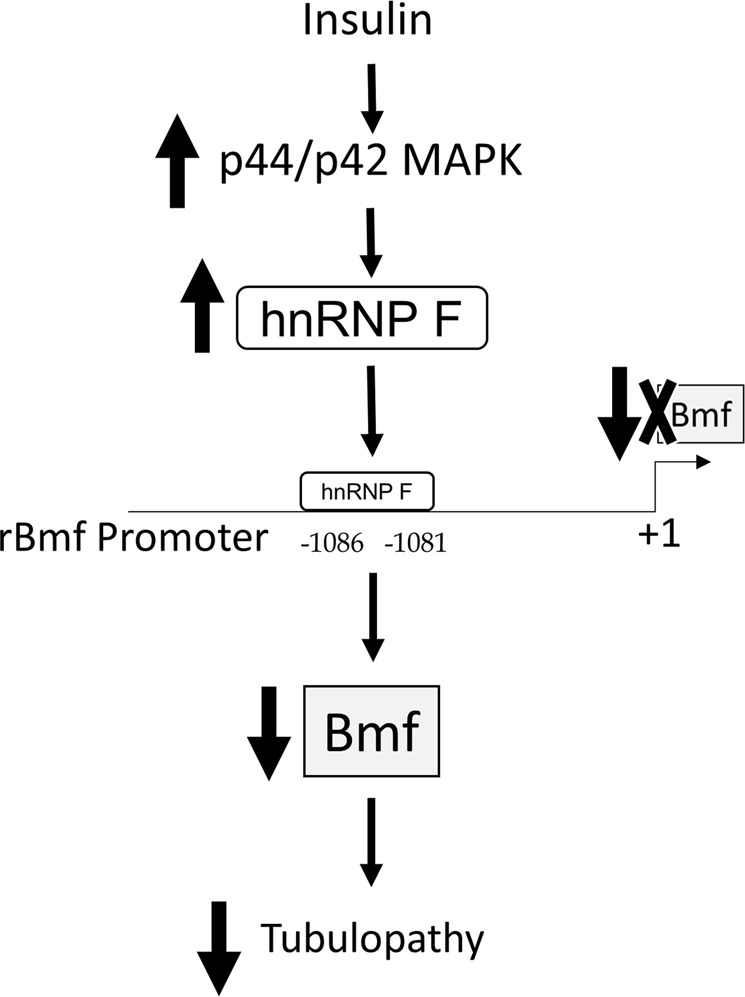
Schematic diagram of mechanism of insulin action on inhibition of Bmf gene transcription and tubulopathy. Insulin stimulates p44/42 MAPK signaling and hnRNP F expression. HnRNP F binds to the hnRNP F-RE (N-1086/N-1081) in the Bmf promoter to inhibit Bmf expression and subsequently prevents Bmf-induction of tubulopathy in diabetic kidney.

In summary, our results demonstrate that insulin inhibits *Bmf* transcription and prevents RPTC apoptosis and loss via p44/42 MAPK signaling and hnRNP F expression (Fig. 8) These findings would imply that Bmf activation could aggravate tubulopathy via inducing RPTC loss in the diabetic kidney. However, it remains to be seen whether renal hnRNP F might be a potential target for the prevention of tubulopathy in the diabetic kidney.

## Methods

### Chemical and reagents

D-glucose, D-mannitol, human insulin, PD98059 [a p44/42 MAPK inhibitor] and wortmannin (a PI-3K inhibitor) were procured from Sigma-Aldrich Canada Ltd. (Oakville, ON, Canada). U0126 (an inhibitor of p44/42 MAPK) was purchased from Cell Signaling Technology (New England BioLabs Ltd., Whitby, ON, Canada). Dulbecco’s modified Eagle medium (DMEM, 5 mmol/L D-glucose, catalog no. 12320), penicillin/streptomycin and fetal bovine serum (FBS) were bought from Invitrogen, Inc. (Burlington, ON, Canada). Insulin implants (Linßit, with a release rate of approximately 0.1 unit/implant/day for 30 days) and pGL4.20 [Luc/Puro] vector containing luciferase reporter were obtained from Linshin (Scarborough, ON, Canada) and Promega Corporation (Sunnyvale, CA), respectively. The antibodies used are listed in Supplementary Table 1. Rat genomic DNA was used to clone the *Bmf* gene promoter N-1370 to N + 102 by conventional polymerase chain reaction (PCR) with specific primers (Supplementary Table 2) and confirmed by DNA sequencing. The *Bmf* gene promoter then was inserted into pGL4.20 vector via Xho I and Hind III enzyme restriction sites. Scrambled Silencer Negative Control small interfering RNA (siRNA), siRNAs of *p44/42 MAPK* and *hnRNP F* were obtained from Ambion, Inc. (Austin, TX). Oligonucleotides and biotin-labeled primers were sourced from Integrated DNA Technologies (Coralville, IA). Kits for LightShift Chemiluminescent electrophoretic mobility shift assay (EMSA) and QuickChange II Site-Directed Mutagenesis were purchased from Agilent Technologies (Santa Clara, CA) and Thermo Scientific (Life Technologies Inc., Burlington, ON, Canada), respectively. Restriction and modifying enzymes were from New England BioLabs, Invitrogen, Roche Biochemicals, Inc. (Dorval, QC, Canada), and GE Healthcare Life Sciences (Baie d’Urfé, QC, Canada).

### Generation of human BMF-Tg and akita hnRNP F-Tg mice

Tg mice specifically overexpressing human *myc*-*BMF* in their RPTCs were generated using a similar strategy to that described previously^20,28,49^. In brief, full-length human *BMF* cDNA fused with Myc-tag was inserted into pKAP2 plasmid at the NotI site. The plasmid pKAP2 containing the KAP promoter that is responsive to androgen was a gift from Dr. Curt D. Sigmund (University of Iowa, Iowa City, IA)^50^.

Akita *hnRNP F*-Tg mice were generated by cross-breeding *hnRNP F*-Tg mice with heterozygous Akita (C57BL/6-Ins2 Akita/J) mice (Jackson Laboratory, Ann Harbor, ME) as previously described^28^.

### Physiological studies

Adult male non-Tg littermates (wild type (WT)) and *myc*-h*BMF*-Tg mice were studied at the age of 10 to 20 weeks. Akita mice and Akita *hnRNP F*-Tg mice were studied at the age of 10 to 16 weeks. Male non-Akita littermates (controls) and Akita mice at the age of 10 weeks treated ± insulin implants and studied from week 12 until week 16^41^. All animals had access to water and standard mouse chow ad libitum. Animal care and procedures followed the Principles of Laboratory Animal Care [National Institutes of Health (NIH) publication no. 85–23, revised 1985: http://grants1.nih.gov/grants/olaw/references/phspol.htm] and were approved by the Centre de recherche du centre hospitalier de l’Université de Montréal (CRCHUM) Animal Care Committee.

Mouse blood glucose levels after 4 to 5 h of fasting were measured by Accu-Chek Performa (Roche Diagnostics, Laval, QC, Canada). Morning SBP was monitored at least 2–3 times per week in each animal for 6 or 10 weeks with Visitech Systems BP-2000 tail-cuff (Apex, NC)^28,30,31,47,51^. The mice were acclimatized to the procedure for at least 15–20 min per day for 5 days before the first SBP measurements.

All animals were individually housed in metabolic cages for 8 h during day time for urine collection and blood was collected by cardiac puncture (centrifuged to obtain serum) before euthanasia at 16 or 20 weeks old. Urine samples were assayed for ACR by enzyme-linked immunosorbent assay (ELISA) (Albuwell and Creatinine Companion, Exocell, Inc., Philadelphia, PA). Immediately after euthanasia, the kidneys were removed, decapsulated, and weighed. Left kidneys were processed for histology and immunostaining and right kidneys for RPT isolation by Percoll gradient^28,30,31,47,51^. Aliquots of freshly isolated RPTs from individual mice were processed for isolation of total RNA and protein.

Fluorescein isothiocyanate-labeled inulin was used to estimate the glomerular filtration rate (GFR) as recommended by the Animal Models of Diabetic Complications Consortium (http://www.diacomp.org/), with slight modifications^28,30,31,47,51^.

In a separate series of studies, conscious male C57Bl/6 mice (aged 12 to14 weeks) after a 4-hour food restriction were used for hyperinsulinemic-euglycemic clamp experiments as previously described^31^.

### Morphologic studies

Kidney sections of 4 µm thick from five to six animals/group were assessed by standard periodic acid–Schiff (PAS) or Masson’s trichrome staining. Immunohistochemistry staining (IHC) was performed by the standard avidin-biotin-peroxidase complex method (ABC Staining, Santa Cruz Biotechnology, Santa Cruz, CA). Semi-quantitation of the relative staining was done by NIH Image J software (http://rsb.info.nih.gov/ij/).

Tubular injury score, mean glomerular tuft, tubular luminal area, and RPTC volumes were assessed on PAS–stained sections at ×200 magnification as described previously^28,30,31,47,51^. Briefly, PAS stained images (8–10 fields/kidney) were assessed for tubular injury in the cortex area that displayed tubular dilation, tubular atrophy, cast formation, vacuolization, degeneration, and loss of the brush border. The tubules were evaluated according to the following injury grade (0–3): 0 = no tubular injury, 1 = less than 25% tubules injured, 2 = 25–50% injured, 3 = more than 50% tubules injured^52^. The score corresponding to tubular injury was calculated for each group by summing and then averaging the grades for each field.

The mean glomerular volume (V_G_) was determined by the method of Weibel^53^ with the aid of an image analysis software system (Motics Images Plus 2.0, Motic, Richmond, BC, Canada). The V_G_ was estimated by the mean glomerular tuft area (A_*T*_) derived from the light microscopic measurement of 30 random sectional profiles of glomeruli from each group (n = 6 animals per group) using the formula: V_*g*_ = β/*k x* A_*T*_^1.5^, where β = 1.382 (shape coefficient for spheres) and *k* = 1.1 (size distribution coefficient).

Tubular luminal areas were measured on renal sections (six animals/group; 4 to 5 sections per kidney, 4 random fields per section, 50 tubules around the glomerulus per field) with the same Motics Image Plus 2.0 image analysis software.

Outer cortical RPTs with similar cross-sectional views and clear nuclear structure were selected for cell volume measurement. Mean cell volume from 100 RPTCs was estimated by the Nucleator method^54^.

### Western blotting

Western blotting (WB) was performed as described previously^28,30,31,47,51^. The relative densities of bands of Bmf, caspase-3, cleaved (active) caspase-3, Bax, Bcl2 and β-actin were quantified by NIH ImageJ software (http://rsb.info.nih.gov/ij/).

### Real-Time quantitative PCR

Real-time quantitative (q) PCR was used to quantify the mRNA levels of various genes in RPTs with the forward and reverse primers listed in Supplementary Table 2 ^28,30,31,47,51^.

### Cell culture

Rat IRPTCs (passages 13 through 18) were used^55^. Rat *Bmf* gene promoter inserted into the plasmid pGL4.20 was stably transfected into IRPTCs as described previously^28,30,31,47,51^.

To investigate insulin effect, IRPTC stable transformants (75% to 85% confluence) were synchronized for 16 hrs in serum-free DMEM (5 mmol/L D-glucose). Then they were incubated in normal glucose (NG, 5 mmol/L D-glucose plus 20 mmol/L D-mannitol) or HG (25 mmol/L D-glucose) DMEM containing 1% depleted fetal bovine serum (FBS) and insulin (10^−7^ mol/L or 573 ng/mL) for up to 24 hours ± PD98059 or U0126 or wortmannin^31^. The cells were then harvested, and the *Bmf* gene promoter activity was measured by luciferase assay^31^. IRPTCs stably transfected with pGL4.20 served as controls. In addition, scrambled siRNA or siRNAs of *p44/42 MAPK* or *hnRNP F* were transfected into IRPTC stable transformants and cultured for 24 hours, their effects on *Bmf* gene promoter activity and mRNA expression were assessed.

### Flow cytometry analysis of urinary cells

Urine was collected for 6 hrs from individual non-Tg or myc-h*BMF-*Tg mice placed individually in metabolic cages. Urines from 5 mice in each group were collected, pooled and centrifuged at 500 × g for 10 minutes to pellet down the cells in the urine. Pellets were rinsed with PBS and passed through a 35 μm cell strainer (#352235, Corning Inc., Corning, USA). Each cell suspension was then blocked with 2% FBS in PBS for 10 minutes then stained with DRAQ7 and anti-prominin-1 antibody conjugated with PE-Vio770 (MiltenyiBiotec, BergischGladbach, Germany, 1:25 dilution)^34^ for 30 minutes at room temperature in the dark, then washed with PBS; equal volumes of suspension from non-Tg or myc-h*BMF-*Tg mice were subjected to flow cytometry (LSR-II, BD Biosciences). Staining with DRAQ7 was used to assess viability. The data was analyzed using FlowJo V10 (FlowJo, LLC, Ashland, USA).

### Statistical analysis

Data are expressed as means ± SEM. Statistical analysis was performed with the Student *t* test or one-way ANOVA and the Bonferroni test, as appropriate (GraphPad Prism 5.0 software, http://www.graphpad.com/prism/Prism.htm). *P* < 0.05 was considered to be statistically significant for all tests. (*p ≤ 0.05; **p ≤ 0.01; ***p ≤ 0.001; NS, non-significant)

## Supplementary information

Supplementary Info
