# Supplementary material for: Heterogeneous Nuclear Ribonucleoprotein F Mediates Insulin Inhibition of Bcl2-Modifying Factor Expression and Tubulopathy in Diabetic Kidney
Source: Sci Rep. 2019 Apr 30;9:6687. doi: 10.1038/s41598-019-43218-2 (PMC6491582; doi:10.1038/s41598-019-43218-2)
Supplement: Supplementary file 1 — Supplementary Info [file 41598_2019_43218_MOESM1_ESM.pdf]

Supplementary Material

Heterogeneous Nuclear Ribonucleoprotein F Mediates Insulin Inhibition of Bcl2-Modifying Factor Expression and Tubulopathy in Diabetic Kidney

Anindya Ghosh[1], Shuiling Zhao[1], Chao-Sheng Lo[1], Hasna Maachi[1], Isabelle Chenier[1], Muhammad Abdul Lateef[1], Shaaban Abdo[1], Janos G. Filep[2], Julie R. Ingelfinger[3], Shao-Ling Zhang[1*] and John S.D. Chan[1*]

[1]Département médecine
Université de Montréal
Centre de recherche du Centre hospitalier de l'Université de Montréal (CRCHUM)
900 Saint Denis Street, Montréal, QC
Canada H2X 0A9

[2]Département de pathologie et biologie cellulaire
Université de Montréal
Centre de recherche, Hôpital Maisonneuve-Rosemont
5415 boul. de l'Assomption, Montréal, QC
Canada H1T 2M4

[3]Harvard Medical School
Pediatric Nephrology Unit
Massachusetts General Hospital
15 Parkman Street, WAC 709, Boston, MA
USA 02114-3117

[1*]John S.D. Chan and Shao-Ling Zhang are joint senior authors to whom correspondence should be addressed.
Telephone: (514) 890-8000 Extension 15080 or 15633, Fax: (514) 412-7655
E-mail: john.chan@umontreal.ca and shao.ling.zhang@umontreal.ca

Supplementary Table 1: Antibodies

Protein Target	Name of Antibody	Manufacturer, Catalog, and/or Name of Individual Providing the Antibody	Species Raised in; Monoclonal or Polyclonal	Dilution for WB and or IHC
Bmf	Bmf antibody	N-19, sc-20181, Santa Cruz Biotechnology	Goat; polyclonal	IHC: 1:200
	Bmf antibody	9G10, Enzo Life Sciences	Rat; monoclonal	WB; 1:2000
	Bmf antibody	Abcam, Ab9655	Rabbit; polyclonal	IP: 1:100
HnRNP F	hnRNP F antibody	Specifically recognizing (CTARRYIGIVKQAGLER) were generated in our laboratory	Rabbit; polyclonal	WB; 1:10000<br>IHC; 1:200<br>EMSA;1-2 μg
AQP 1	AQP 1 antibody	H-55, sc-20810	Rabbit; polyclonal	WB; 1:2000
	AQP 1 antibody	1/22, sc-32737	Mouse; monoclonal	IF; 1:200
TGFβ	TGFβ antibody	Sc-146, Santa Cruz Biotechnology	Rabbit polyclonal	WB; 1:2000
Bax	Bax antibody	P-19, sc-526, Santa Cruz Biotechnology	Rabbit polyclonal	WB; 1:2000
	Bax antibody	B-9, sc-7480, Santa Cruz Biotechnology	Mouse monoclonal	WB; 1:2000<br>IP; 1:100
Bcl-2	Bcl-2 antibody	C-2, SC-7382, Santa Cruz Biotechnology	Mouse; monoclonal	WB; 1:2000
	Bcl-2 antibody	D17C4, 3498S, Cell signaling	Rabbit; monoclonal	IP; 1:100
Caspase 3	Caspase 3 antibody	9662S, Cell signaling	Rabbit; polyclonal	WB; 1:2000
Cleaved caspase 3	Cleaved caspase 3 antibody	9661S (ASP175), Cell signaling	Rabbit; polyclonal	WB; 1:2000
β-Actin	β-Actin antibody	AB_476744, Sigma-Aldrich	Mouse; Monoclonal	WB; 1:20000
Prominin-1	PE-Vio-770 Prominin-1 antibody	Miltenyi Biotec, Clone: MB9-3G8	Rat; polyclonal	FACS; 1:25
c-Myc	c-Myc antibody	9E10, sc-40, Santa Cruz Biotechnology	Mouse; monoclonal	WB; 1:2000

Supplementary Table 2: Primers and siRNA

Gene	Sequence	Species	Reference Sequence
BMF	F: CCCTTGGGGAGCAGCCCCCTG<br>R: GCCGATGGAACTGGTCTGCAA	Mouse	NM_139258
BMF	F: GATTGTCCCCTCAGTCGGCT<br>R: CTCGGTGCTGAAGGAACTGTC	Rat	NM_139258.2<br>NM_139258.3
Myc-hBMF	F: ATGGCATCAATGCAGAAGCTGATCTCAGAG<br>R1 (690bp): GCAGAGAGGTTTGCCTTACC<br>R2 (187bp): GTTTGCCCAGAGCCTACTGG<br>R3 (325bp): CACAAGGCAGCATGACACC	Human -Trans-gene	Detects Myc tagged human Bmf cDNA NM_001003943.1
TGF-β1	F: CCAAACTAAGGCTCGCCAGTC<br>R: GGCACTGCTTCCCGAATGTC	Mouse	NM_011577.1
Col1α	F: ATCTCCTGGTGCTGATGGAC<br>R: ACCTTGTTTGCCAGGTTCA	Mouse	NM_007742.3
FN1	F: GGCCTGAACCAGCCTACAG<br>R: TGAGCTTAAAGCCAGCGTCA	Mouse	NM_010233.2
Bax	F: GTTTCATCCAGGATCGAGCAG<br>R: CATCTTCTTCCAGATGGTGA	Mouse<br>Rat	NM_007527.3<br>NM_017059.2
Bcl-2	F: CTGTGGATGACTGAGTACC<br>R: GAGACAGCCAGGAGAAAT	Mouse<br>Rat	NM_009741.5<br>NM_016993.1
hnRNP F	F: AGAGTGACCGGAGAAGCTGA<br>R: GCTCTCCAGGCCACTGTAAG	Mouse<br>Rat	NM_133834.2<br>NM_001037286.1
β-actin	F: ATGCCATCCTGCGTCTGGAC<br>R: AGCATTTGCGGTGCACGATGG	Mouse<br>Rat	NM_007393.3<br>NM_031144.2
Rat Bmf Promoter	F: AAAAAACTCGAGCCTCGTTTTCCCCCAAATCCATC<br>F: GGCAAACTCGAGGACCCAGGTTCAATTCC<br>F: GGCAAACTCGAGCTGACCTAAATCTACCTG<br>F: GACCTGCTCGAGTACTTTATTC<br>F: GCTTTCTGGAAGTCCCGCA<br>R: AAAAAAAGCTTCTGCGGTGGGAGGTGGTG	Rat	-1370 Xho 1<br>-1260 Xho1<br>-1045 Xho1<br>-965 Xho1<br>-351 Xho1<br>+102 HindIII
Site directed mutagenesis primers for Rat Bmf Promoter hnRNP F binding element	F: GAGGATGCCTGATAGCTCAATACTGACCTTGCTT<br>R: AAGCAAGGTCAGTATTGAGCTATCAGGCATCCTC<br>F: CCTCATCCTGTTATTCCTACCTCTGCAATCTGACC<br>R: GGTCAGATTGCAGAGGTAGGAATAACAGGATGAGG<br>F: GACCAGCAACTCCTTTTTTTATACAAGTCGCGGCT<br>R:AGCCGCGACTTGTATAAAAAAAGGAGTTGCTGGTC	Rat	Bmf (-1370/+102) Δ997-991<br>Bmf (-1370/+102) Δ1086-1081<br>Bmf (-1370/+102) Δ402-395
Rat Bmf promoter hnRNP F-RE probe	F: TTATTCCAGGGGGTACCTCTG<br>R: CAGAGGTACCCCCTGGAATAA		Biotinylated probe for EMSA (-1086)
HnRNP F RE (WT)	F: TTATTCCAGGGGGTACCTCTG<br>R: CAGAGGTACCCCCTGGAATAA		competitor
HnRNP F RE (Mut 1)	F: TTATTCCAaaaaaTACCTCTG<br>R: CAGAGGTAtttttTGGAATAA		competitor
HnRNP F RE (Mut 2)	F: TTATTCCAGGaaaTACCTCTG<br>R: CAGAGGTAtttCCTGGAATAA		competitor
HnRNP F RE (Mut 3)	F: TTATTCCAaaGGGTACCTCTG<br>R: CAGAGGTACCCttTGGAATAA		competitor
si hnRNP F (ID:s133896)	F: CAUGCAGCACAGAUACAUAtt<br>R: UAUGUAUCUGUGCUGCAUGtt	Rat	siRNA

Supplemental Figure 1

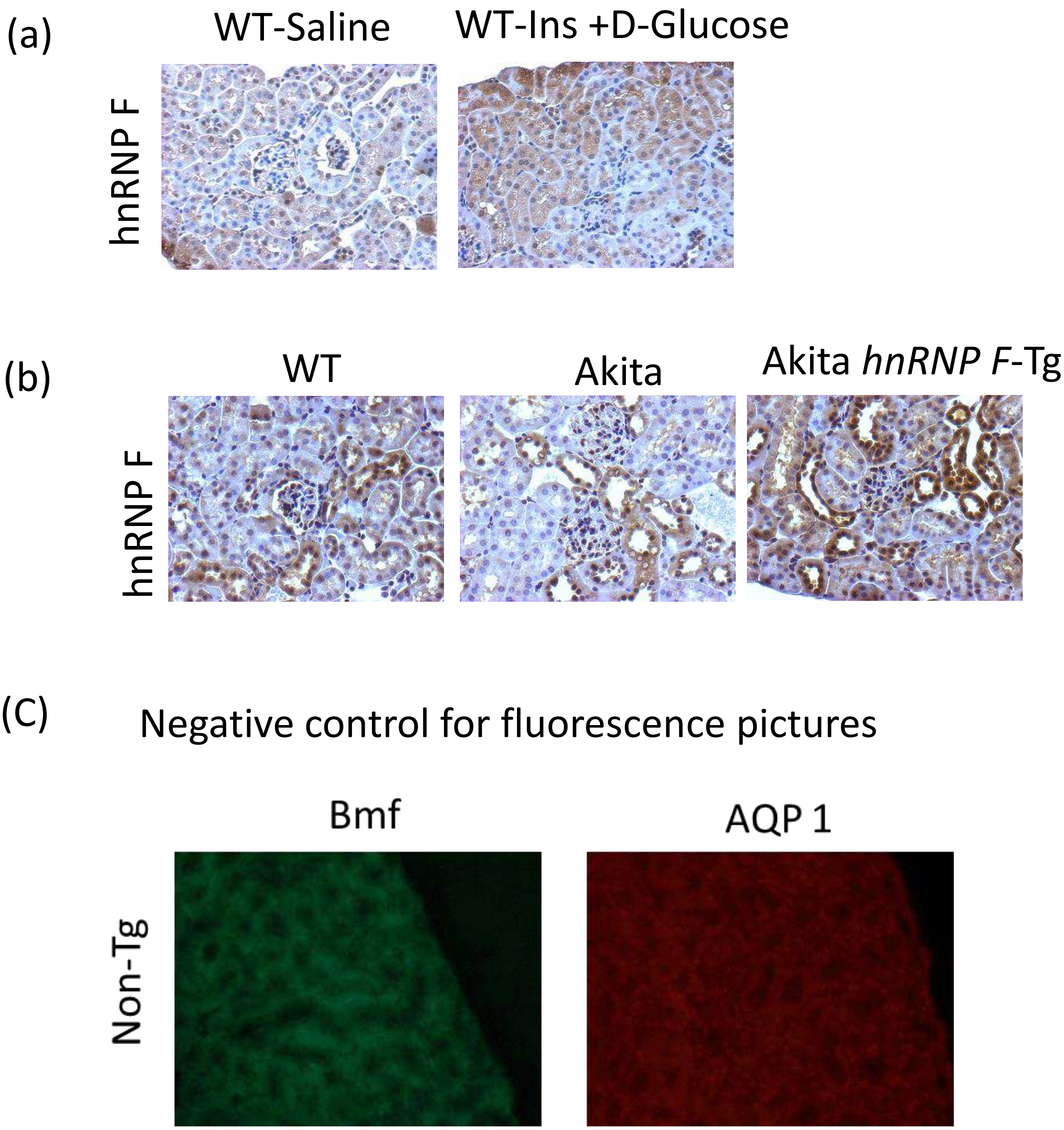

Figure S1: Immunohistochemical staining for hnRNP F levels in the RPTs from (a) WT mice after 3-h infusion with saline or insulin (ins) and (b) WT, Akita and Akita *hnRNP F*-Tg mice. (magnification X 200). (c) Negative control for fluorescence pictures in figure 1 (g) where primary antibodies were omitted from the reaction.

Original Blots

1b.

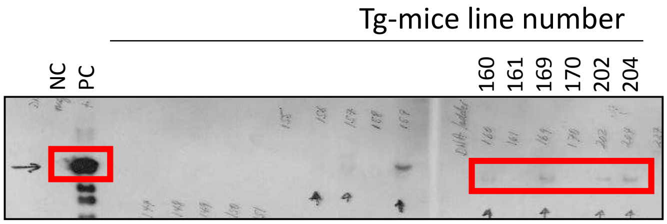

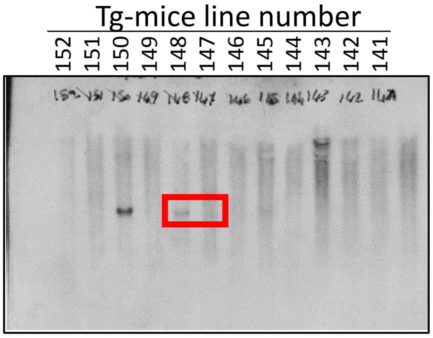

Founder F0: Southern blotting of genomic DNA for founders with biotin-labeled *BMF* probe. PC, plasmid positive control. NC, negative control.

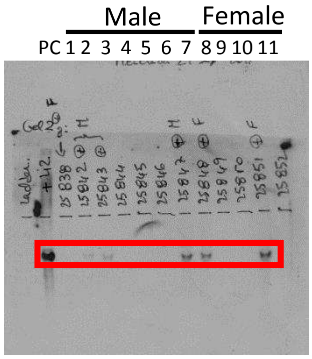

Line 148-F1: Southern blotting of genomic DNA for F1 generation

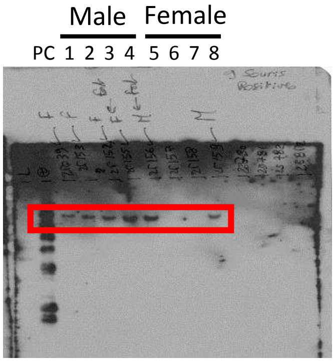

Line 148-F2: Southern blotting of genomic DNAfor F2 generation

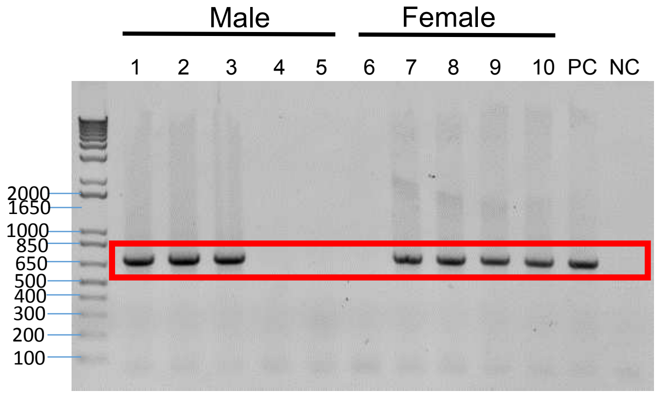

Line 148-F3: PCR to detect hBMF transgene for F3 generation

1c.

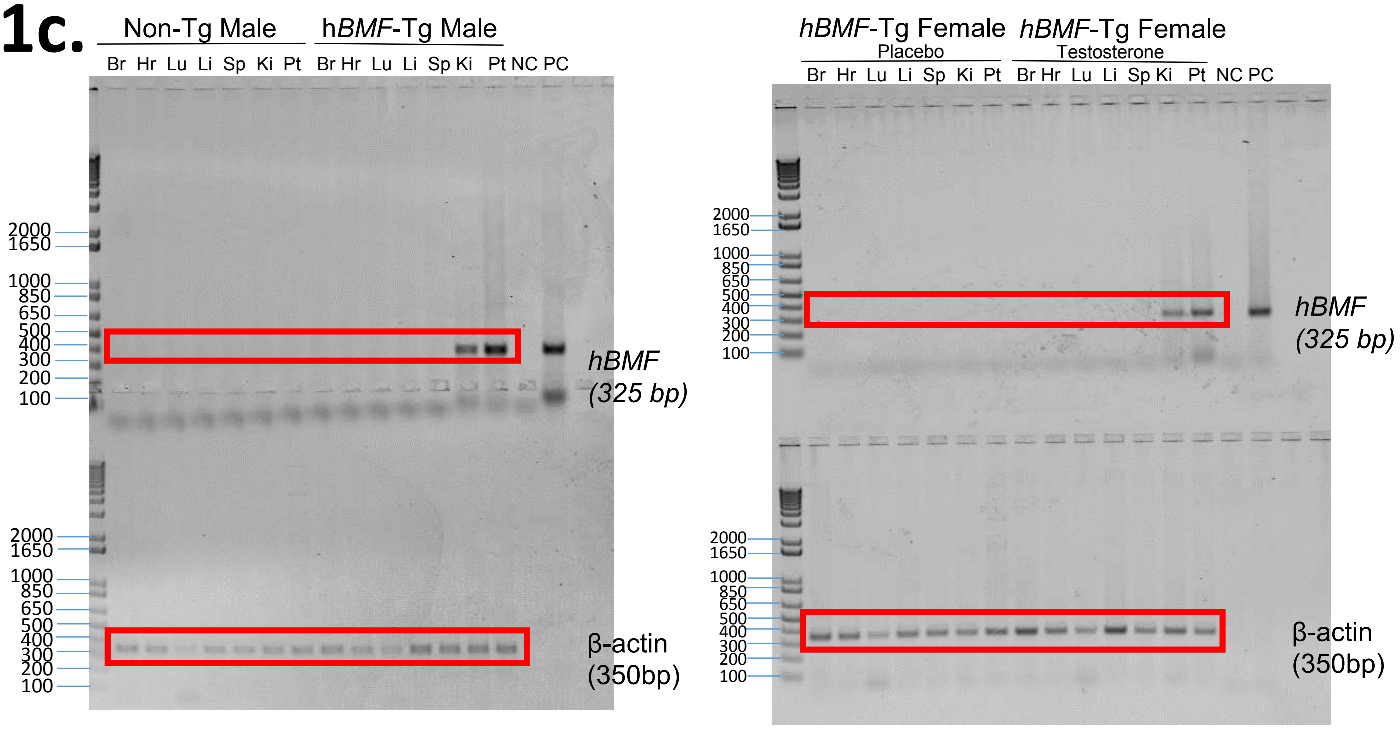

Figure 1c. RT-PCR product showing tissue expression of *hBMF* mRNA in male and in female Tg mice un-induced or induced with testosterone. *hBMF* and β-*actin* fragments are indicated. Female transgenic mice (line #148) mice were induced with placebo pellets or pellets containing 5 mg testosterone with a 21-day release schedule (Cat. #A-121, Innovative Research of America, Sarasota, FL) for 2 weeks prior to RNA isolation. Br, brain; Hr, heart; Lu, lung; Li, liver; Sp, spleen; Ki, kidney; PT, isolated proximal tubule; PC, Positive control; NC, Negative control.

1d.

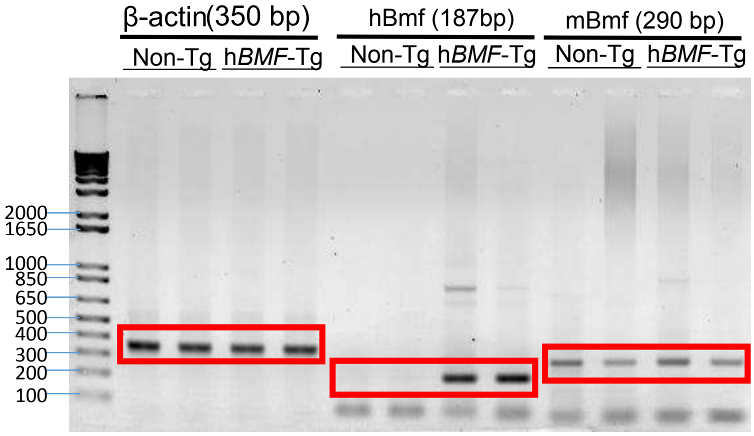

Figure 1d. Specific PCR analysis of *hBMF* transgene and mouse Bmf in offspring of non-Tg and *hBMF*-Tg from line 148.

Additional Supplementary Figures

1e.

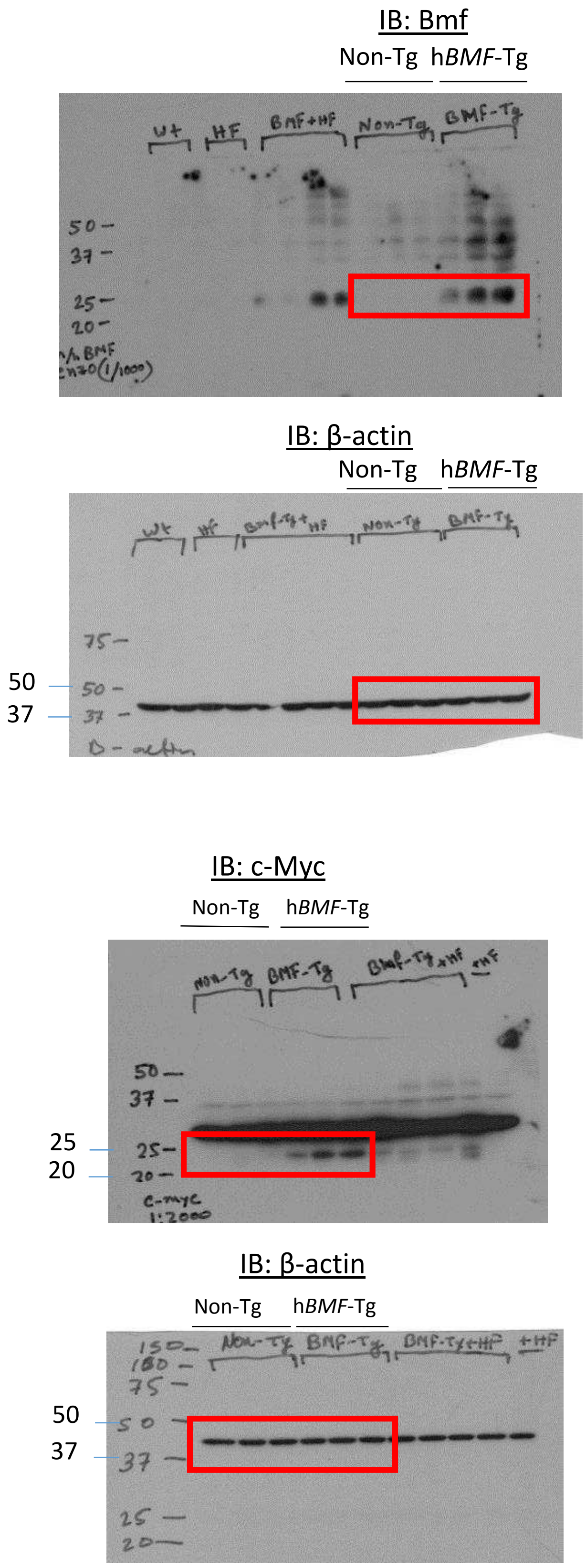

Figure 1e. WB of c-Myc, Bmf and β-actin protein expression in non-Tg and *hBMF*Tg mice.

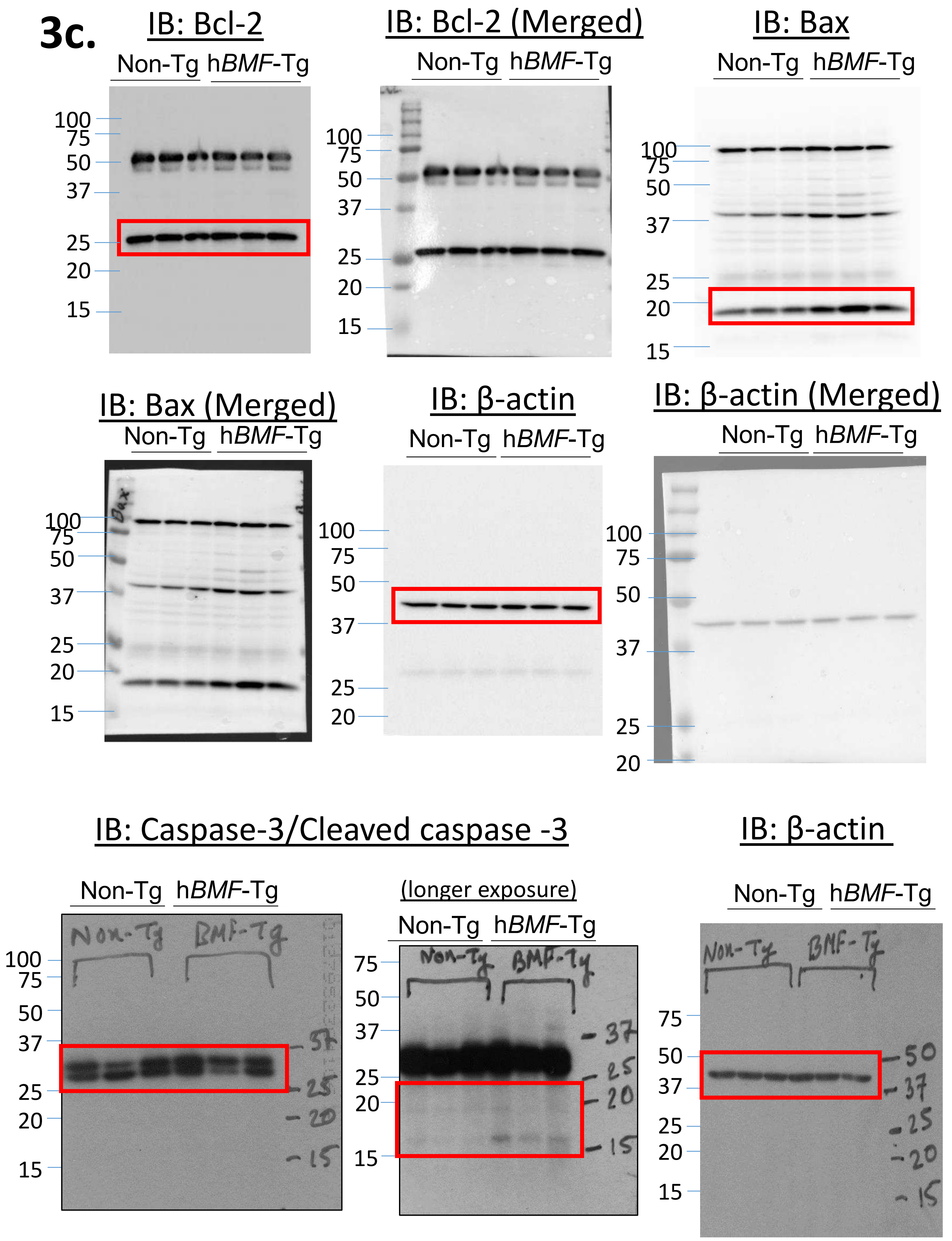

Figure 3c. WB of Bcl-2, Bax, caspase-3 and cleaved -caspase-3 in freshly-isolated RPTs from non-Tg and *hBMF*-Tg mice.

3e. Input

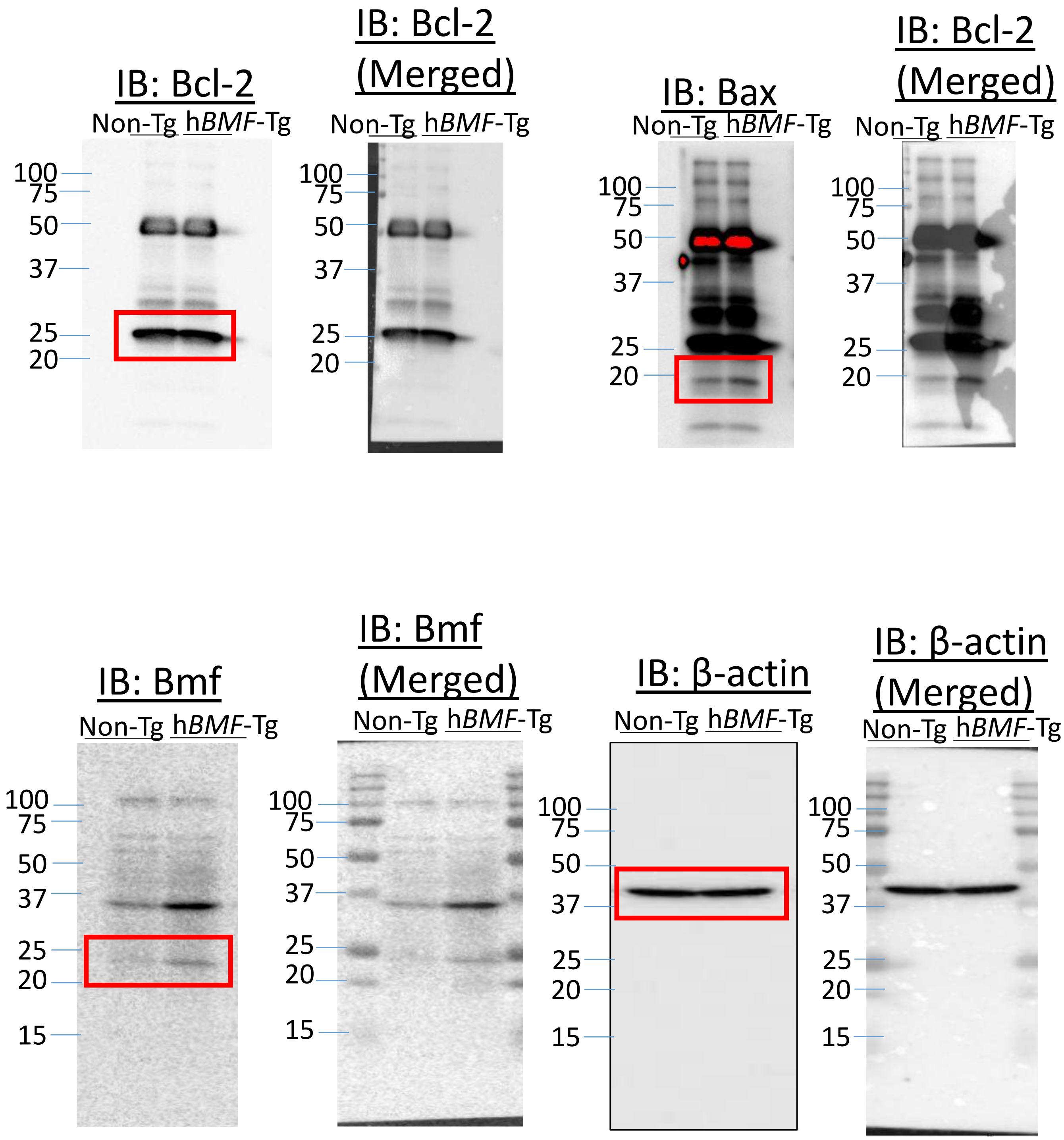

Figure 3e. WB for Bcl-2, Bax, Bmf from total lysate of RPTs (Input) as indicated in Non-Tg or Bmf-Tg animals

3e.

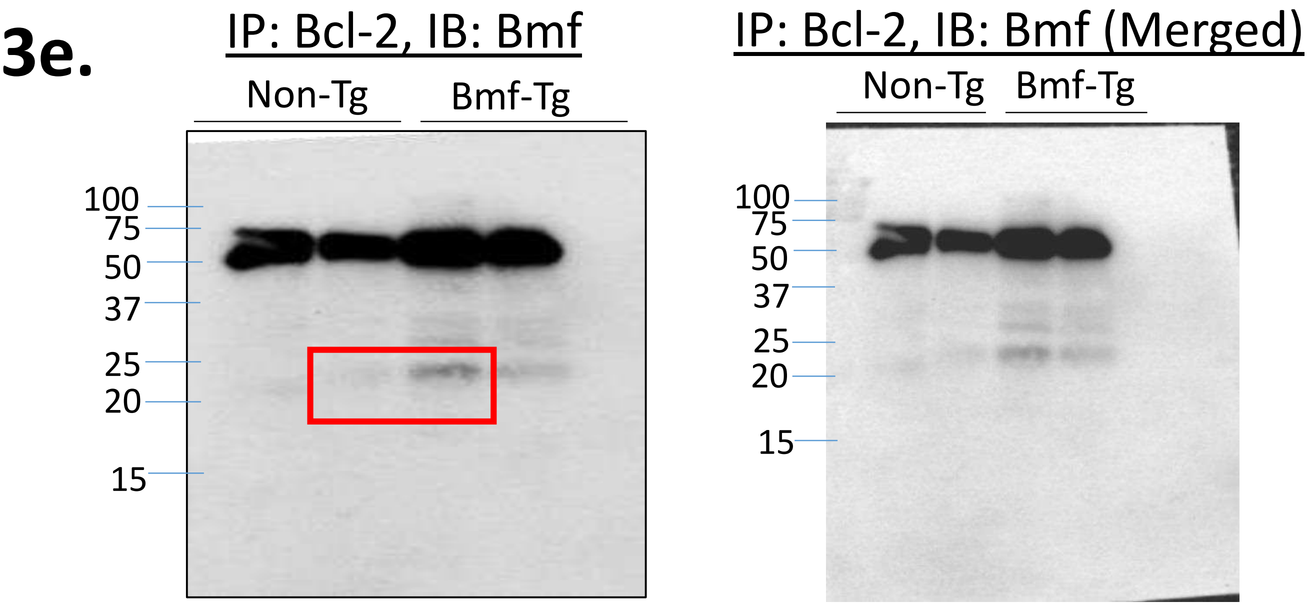

Figure 3e. Co-immunoprecipitation with Bcl-2 and WB with Bmf as indicated in Non-Tg or Bmf-Tg animals.

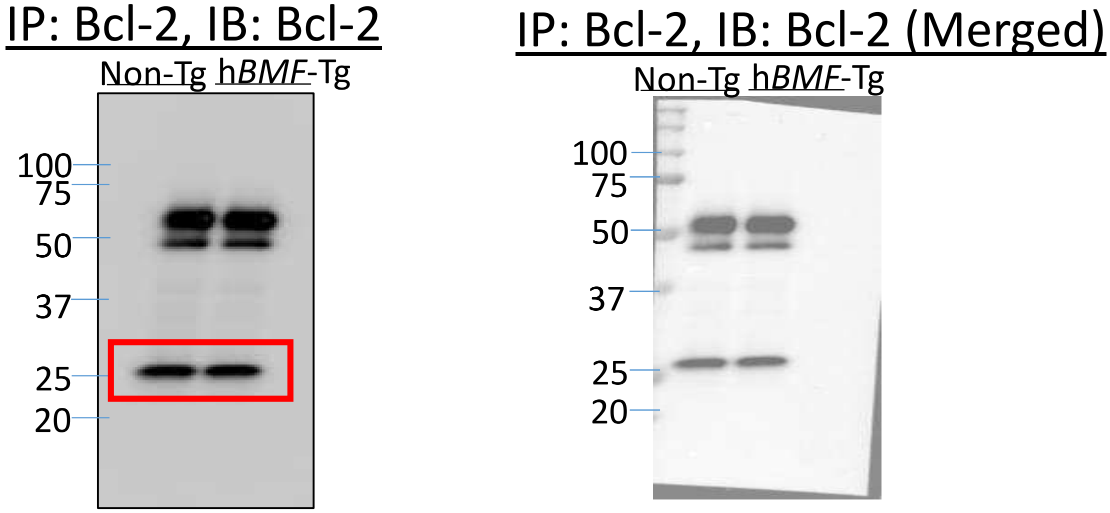

Figure 3e. Co-immunoprecipitation with Bcl-2 and WB with Bcl-2 in Non-Tg or Bmf-Tg animals.

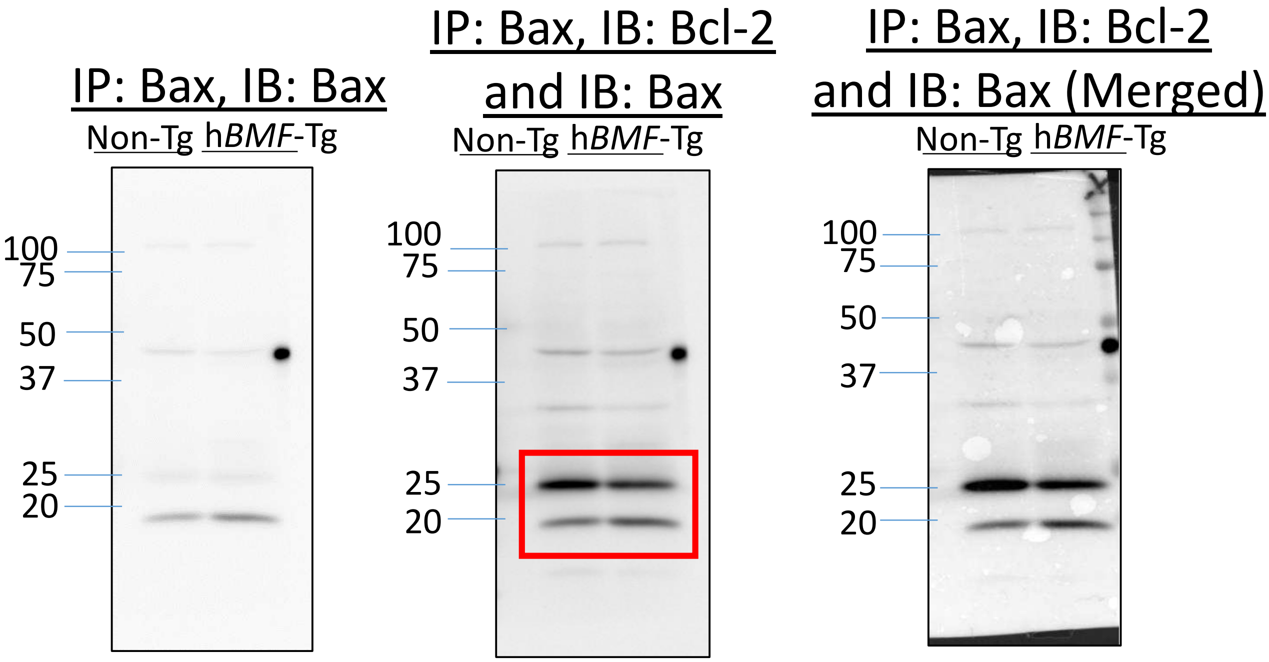

Figure 3e. Co-immunoprecipitation with Bax and WB with Bax and Bcl-2 in Non-Tg or Bmf-Tg animals.

Additional Supplementary Figures

4b.

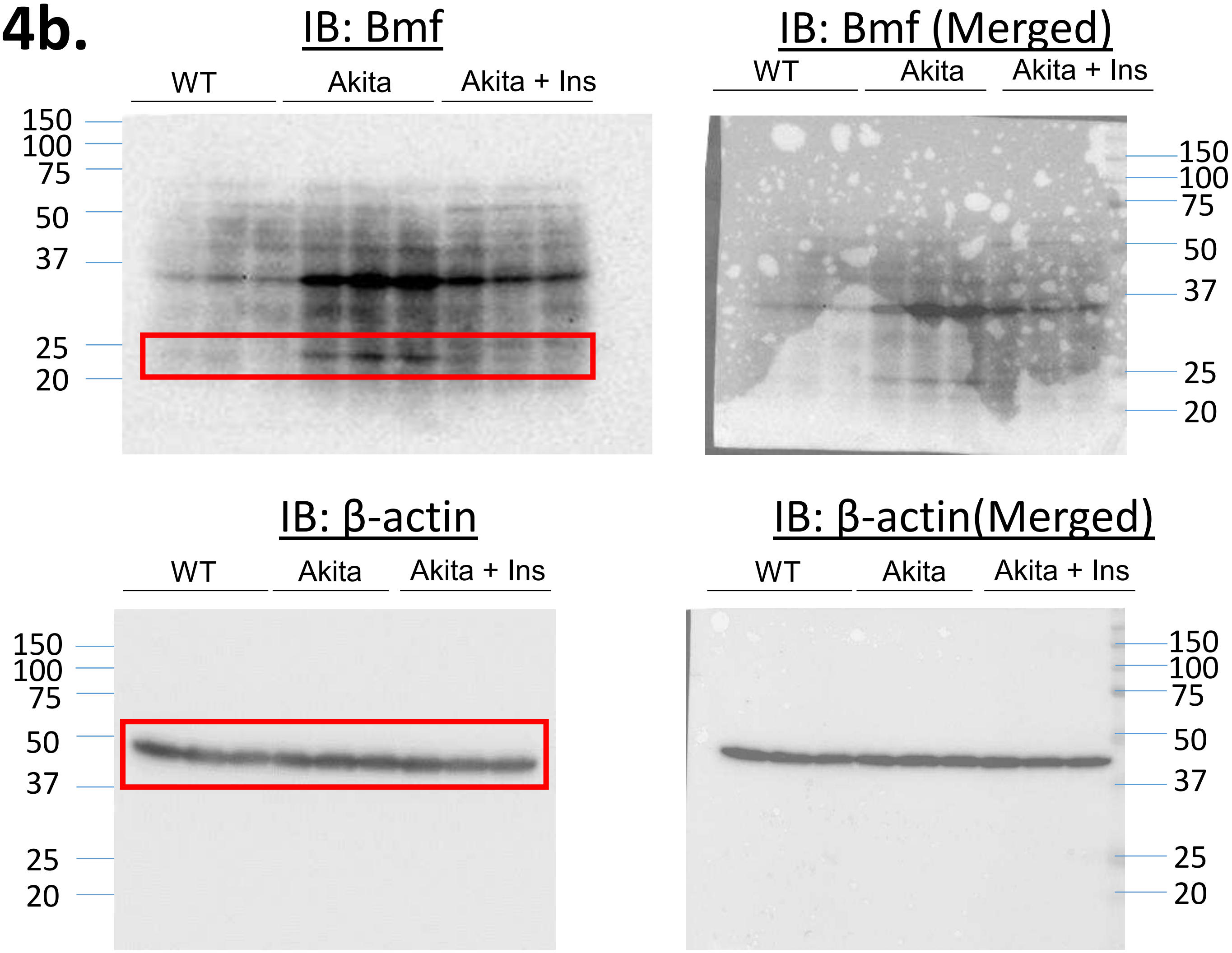

WB of Bmf and β-actin in freshly-isolated RPTs from WT, Akita and insulin-treated Akita mice.

5c.

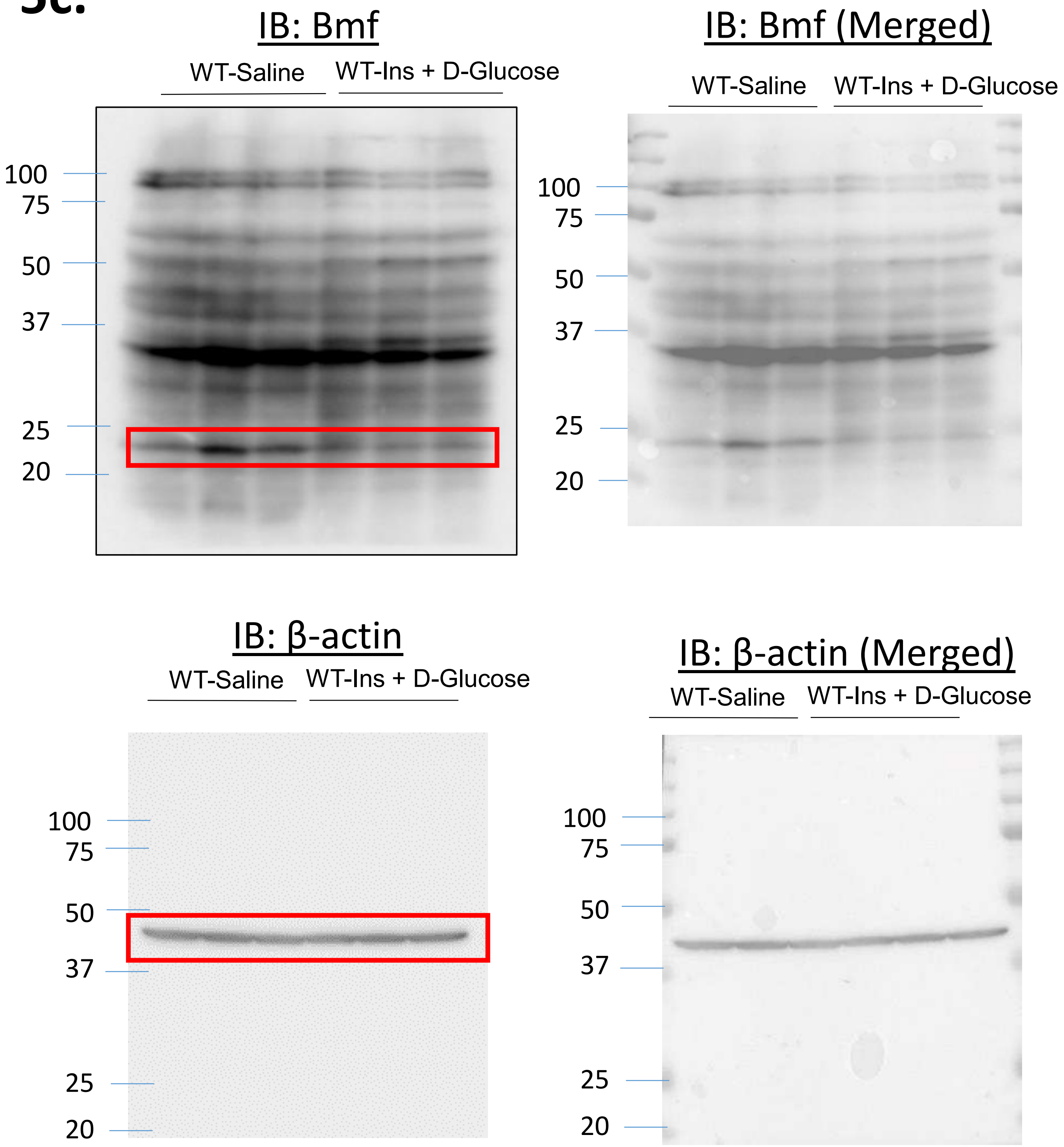

WB of Bmf and β-actin expression in isolated RPTs from WT mice after 3-h infusion with saline or insulin (Ins) + D-glucose .

5e.

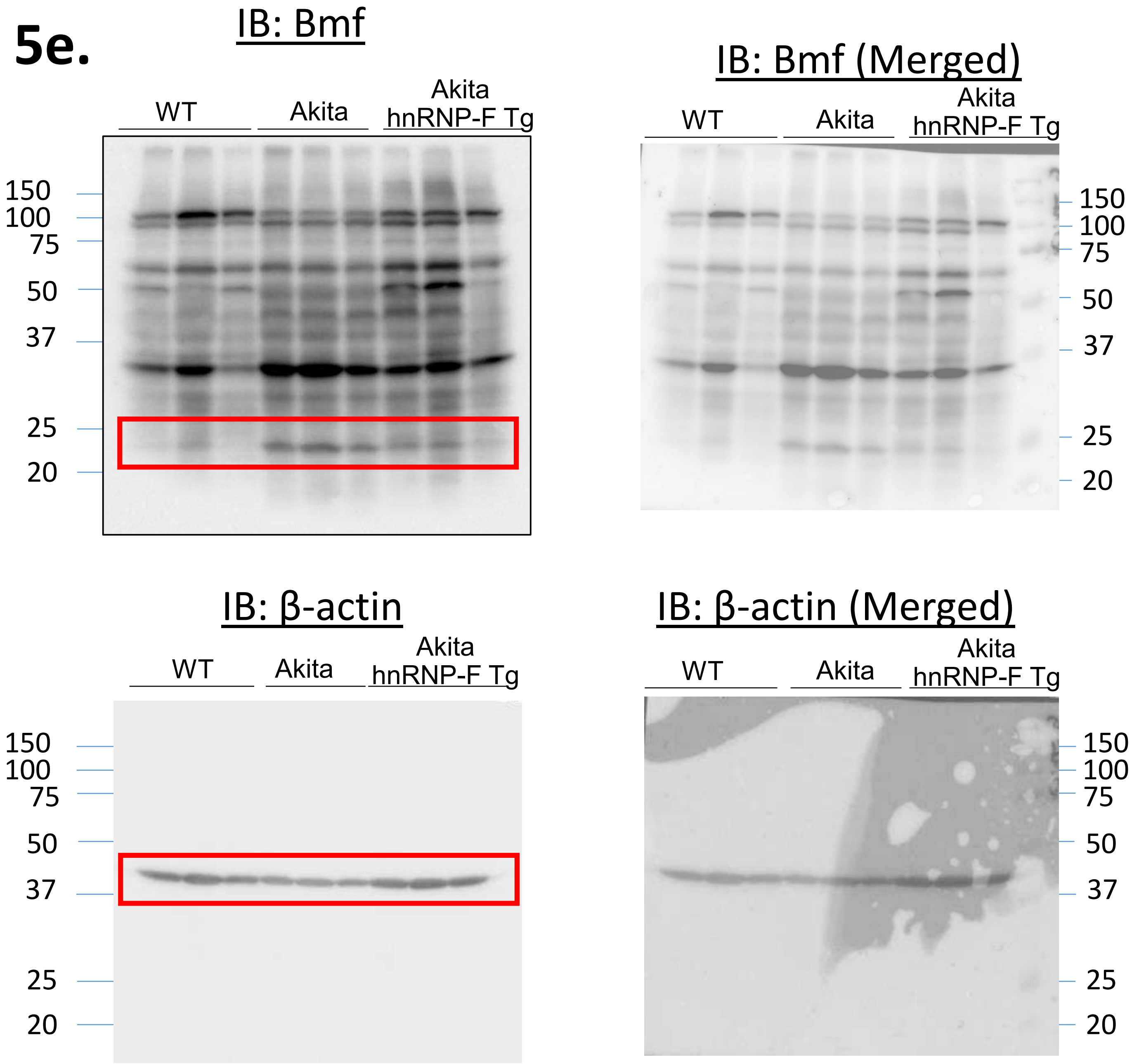

WB of Bmf and β-actin in RPTs from WT, Akita and Akita-hnRNP F-Tg mice

7e. (i)

EMSA

	1	2	3	4	5	6	7	8	9	10
Nuclear extract (5µg)	-	BSA	+	+	+	+	+	+	+	BSA
Biotinylated hnRNP F-RE	+	+	+	+	+	+	+	+	+	+
5′-TTATTCCAGGGGGTACCTCTG-3′(WT)	-	-	-	200x	-	-	-	200x	-	-
5′-TTATTCCAAAAAATACCTCTG-3′(M1)	-	-	-	-	200x	-	-	-	-	-
5′-TTATTCCAGGAAATACCTCTG-3′(M2)	-	-	-	-	-	200x	-	-	-	-
5′-TTATTCCAAAGGGTACCTCTG-3′(M3)	-	-	-	-	-	-	200x	-	-	-

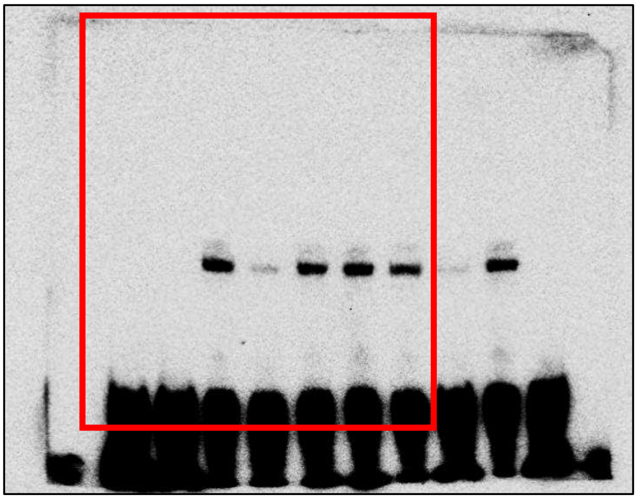

(ii)

	1	2	3	4	5	6	7	8	9	10
Nuclear extract (5µg)	BSA	+	+	+	+	+	+	+	+	+
Biotinylated hnRNP F-RE	+	+	+	+	+	+	+	+	+	+
5′-TTATTCCAGGGGGTACCTCTG-3′(WT)	-	-	200x	-	-	-	-	-	-	-
5′-TTATTCCAAAAAATACCTCTG-3′(M1)	-	-	-	200x	-	-	-	-	-	-
5′-TTATTCCAGGAAATACCTCTG-3′(M2)	-	-	-	-	200x	-	-	-	-	-
5′-TTATTCCAAAGGGTACCTCTG-3′(M3)	-	-	-	-	-	200x	-	-	-	-
Anit-hnRNP F antibody (abcam)(ug)	-	-	-	-	-	-	2	-	-	-
Anti-hnRNP F antibody(µg)	-	-	-	-	-	-	-	1	2	-
Rabbit IgG(µg)	-	-	-	-	-	-	-	-	-	2

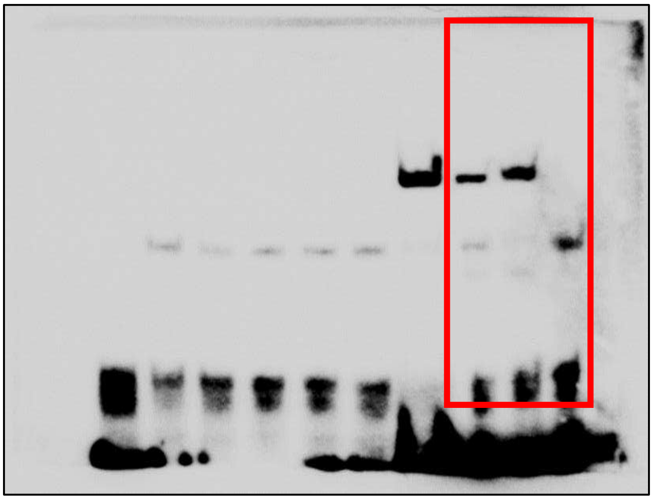

EMSA of putative biotinylated *IRE* (N-1093 to N-1072) with RPTC nuclear proteins with or without excess unlabeled WT *IRE* or mutated *IRE*. For supershift rabbit anti-hnRNP F or rabbit IgG was added to the reaction mixture and incubated for 30 minutes on ice before incubation with biotinylated probe..
